# Genomic characterization of extended‐spectrum beta‐lactamase‐producing and carbapenem‐resistant *Escherichia coli* from urban wastewater in Australia

**DOI:** 10.1002/mbo3.1403

**Published:** 2024-03-15

**Authors:** Zillur Rahman, Mary‐Louise McLaws, Torsten Thomas

**Affiliations:** ^1^ School of Biological, Earth and Environmental Sciences, Centre for Marine Science and Innovation UNSW Sydney Sydney New South Wales Australia; ^2^ School of Population Health UNSW Sydney Sydney New South Wales Australia; ^3^ UNSW Global Water Institute UNSW Sydney Sydney New South Wales Australia

**Keywords:** emerging strain, *Escherichia coli*, novel resistance mechanism, wastewater‐based surveillance, whole‐genome sequencing

## Abstract

This study investigates extended‐spectrum beta‐lactamase‐producing and carbapenem‐resistant *Escherichia coli* isolates from Sydney's wastewater. These isolates exhibit resistance to critical antibiotics and harbor novel resistance mechanisms. The findings highlight the importance of wastewater‐based surveillance in monitoring resistance beyond the clinical setting.

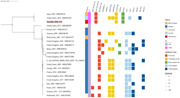

## 1 INTRODUCTION

*Escherichia coli* is a versatile bacterial species in the family *Enterobacteriaceae* and comprises strains that are harmless inhabitants, as well as pathogenic variants capable of causing intestinal or extraintestinal infections in humans and animals (Kaper et al., 2004; Sarowska et al., 2019). *E. coli* strains that are resistant to multiple antibiotic classes, including last‐resort carbapenems and polymyxins, are increasing worldwide (WHO, 2014), leaving fewer or sometimes no effective antibiotics for treatment. This may cause increased hospitalization rates, morbidity, mortality, and treatment costs (Batalla‐Bocaling et al., 2021; Magiorakos et al., 2012).

The most common resistance mechanism in *E. coli* is the production of different β‐lactamases that render these organisms resistant to clinically important β‐lactam antibiotics such as penicillins, cephalosporins, and carbapenems (Nordmann & Poirel, 2019; Peirano & Pitout, 2019). The cefotaximase‐Munich (CTX‐M)‐type extended‐spectrum β‐lactamases (ESBL), cephamycinase (CMY)‐type AmpC β‐lactamases, and imipenemase (IMP)‐, *Klebsiella pneumoniae* carbapenemases‐, New Delhi metallo‐β‐lactamase (NDM)‐, and oxacillinase‐48 (OXA‐48)‐type carbapenemases are the most clinically relevant β‐lactamases associated with healthcare‐ and community‐acquired infections around the globe (Boyd et al., 2020; Bush & Bradford, 2020; Peirano & Pitout, 2019).

The global dissemination of multidrug‐resistant (MDR) *E. coli* is also mediated by a small number of epidemiologically successful clones (Mathers et al., 2015; Woodford et al., 2011). Among these, the sequence type 131 (ST131) is the most prevalent high‐risk clone owing to its worldwide distribution, high level of virulence and fitness, persistence and ease of transmission between human and nonhuman hosts, and resistance to β‐lactams such as third‐generation cephalosporins and fluoroquinolones (Pitout & Finn, 2020). Examples of other clinically significant MDR, high‐risk *E. coli* clones include ST10, ST38, ST69, ST73, ST95, ST155, ST393, ST405, and ST648 (Manges et al., 2019; Riley, 2014). Several recent reports have also documented carbapenemase‐producing ST167, ST410, and fluoroquinolone‐resistant ST1193 as emerging international high‐risk clones (Garcia‐Fernandez et al., 2020; Johnson et al., 2019; Roer et al., 2018).

In Australia, *E. coli* has been considered a priority pathogen of clinical significance, and resistance to common agents used to treat *E. coli* infections has been monitored at the national level using the healthcare‐based Antimicrobial Use and Resistance in Australia (AURA) surveillance system (AURA, 2016). A major limitation of the AURA and other international healthcare infection surveillance is the inability to monitor the emergence and spread of resistance in *E. coli* beyond clinical settings, that is, in the community and nonhuman sources, such as animals and the environment. These nonhuman and community‐based sources have been proposed as potential reservoirs for amplifying and transmitting resistant organisms or genes across different niches (Bailey et al., 2010; Woolhouse et al., 2015). Therefore, it is crucial to monitor antibiotic‐resistant *E. coli* beyond the clinical setting to effectively track and control the emergence, spread, and transmission of the ever‐increasing rate of resistance. As wastewater receives bacteria from humans, animals, and environmental sources, routine wastewater‐based surveillance emerges as a pivotal approach to monitoring the occurrence and diversity of antibiotic‐resistant *E. coli* that might be circulating in the community (Kwak et al., 2015; Reinthaler et al., 2013). Despite its potential, only a limited number of studies, notably from Norway (Jørgensen et al., 2017; Paulshus et al., 2019), England (Raven et al., 2019), Finland (Heljanko et al., 2024), and Uganda (Gomi et al., 2024), have genetically characterized *E. coli* from untreated wastewater to serve as a surrogate indicator to identify dominant and high‐risk lineages, resistance genes, and mobile genetic elements prevalent in the general community.

In our previous study (Rahman et al., 2023), we used wastewater‐based surveillance to monitor antibiotic resistance trends in several priority pathogens in the urban community of Sydney, Australia. From untreated influent wastewater collected at six time points between 2017 and 2019 from 25 Sydney wastewater treatment plants, ESBL‐producing *E. coli* (ESBL‐EC) were consistently detected, and carbapenem‐resistant *E. coli* (CR‐EC) sporadically occurred. Here, we aimed to understand what antibiotic resistance patterns these wastewater *E. coli* isolates from Sydney  have, whether (or not) these isolates are phylogenetically related to any known pandemic high‐risk clones, what molecular mechanisms underpin their resistance, as well as their virulence potential.

## 2 MATERIALS AND METHODS

### 2.1 Bacterial isolation, identification, and antibiotic susceptibility testing

Isolates exhibiting resistance against the largest number of antibiotics that are critically important for human health (WHO, 2019) were selected for this study from a collection of CR‐EC and ESBL‐EC isolates (*n* = 163) (Rahman et al., 2023). Briefly, the collection was generated by serial dilutions of wastewater samples (1:10 and 1:100) that were plated onto ChromID ESBL and CARBA SMART agar (bioMérieux). Pink colonies from both plate types were confirmed as *E. coli* using matrix‐assisted laser desorption ionization‐time of flight mass spectrometry (MALDI‐TOF MS; Biotyper version 3.1, Bruker Daltonics).

The antibiotic susceptibility of these *E. coli* isolates against 18 different antibiotics contained in the AST‐N246 card was determined using a VITEK 2 instrument (bioMérieux, Vitek‐Australia Pty. Ltd.) following the manufacturer's instructions and the Clinical and Laboratory Standards Institute's (CLSI) guidelines (Institute CaLS, 2018). The VITEK 2 advanced expert system™ software (version 8.0) confirmed that suspected pink colonies on ChromID ESBL agar are ESBL‐producing *Enterobacteriaceae* (Blaak et al., 2015) based on the MIC patterns of several β‐lactam antibiotics (i.e., ampicillin, cefazolin, cefoxitin, ceftazidime, ceftriaxone, cefepime, and meropenem) and the β‐lactams with β‐lactamase inhibitors (i.e., amoxicillin‐clavulanic acid, piperacillin‐tazobactam, and ticarcillin‐clavulanic acid). Pink colonies on CARBA SMART agar were confirmed as carbapenem‐resistant based on elevated meropenem resistance (i.e., MIC ≥ 4 µg/mL) in accordance with the CLSI guidelines. Resistance to fosfomycin was determined by the Kirby‐Bauer disk diffusion method (Bauer et al., 1966) using 200 μg fosfomycin plus 50 μg glucose‐6‐phosphate disks (Mast Group Ltd.) according to the CLSI guidelines.

### 2.2 Whole‐genome sequencing, assembly, and annotation

*E. coli* strains were revived from glycerol stocks on horse blood agar plates (bioMérieux), and a single colony was picked and grown in LB broth. Genomic DNA was extracted from the overnight culture using the Monarch® Genomic DNA Purification Kit (New England Biolabs), following the manufacturer's instructions. Sequencing libraries were prepared with the Nextera XT kit, followed by 250 base pair (bp) paired‐end sequencing on the Illumina MiSeq platform at the Ramaciotti Center for Genomics at UNSW Sydney. The raw sequencing reads were quality‐ and adapter‐trimmed using Trimmomatic v.0.38 (Bolger et al., 2014), de novo assembled using the SPAdes genome assembler v.3.15.0 (Bankevich et al., 2012), and annotated using Prokka v.1.14.5 with the default databases (Seemann, 2014).

### 2.3 Bioinformatic and phylogenetic analysis

Sequence types (STs), antibiotic resistance genes, plasmid replicons, and virulence genes of assembled *E. coli* genomes were predicted using MLST 2.0 (Larsen et al., 2012), ResFinder 4.0 (Bortolaia et al., 2020), PlasmidFinder 2.1 (Carattoli et al., 2014), and VirulenceFinder 2.0 (Joensen et al., 2014), respectively, using a minimum nucleotide identity of 90% and a minimum alignment coverage of 60%. Antibiotic resistance or virulence genes are considered plasmid‐borne if located on a contig with plasmid replicons.

The maximum likelihood phylogeny of the *E. coli* genomes was inferred from an alignment of concatenated MLST genes (*adk–fumC–gyrB–icd–mdh–purA–recA*), produced using MAFFT v.7.407 (Katoh & Standley, 2013), applying the general time reversible (GTR)‐Gamma model with 1000 bootstraps in RAxML v.8.2.10 (Stamatakis, 2014). To contextualize the broader relevance of wastewater isolates, a total of 1036 random *E. coli* genomes belonging to similar ST complexes from different countries, sources, and years were retrieved from the Enterobase database (Zhou et al., 2020). Maximum‐likelihood phylogenetic trees were constructed from the alignments of the concatenated core coding sequence (CDS) created using the ‐mafft option in Roary v.3.12.0 (Page et al., 2015) and RAxML with the GTR‐Gamma model. Phylogenetic trees were visualized with metadata in iTOL v.6.3 (Letunic & Bork, 2021).

Mutations were predicted from read alignments of the study isolates with closely related reference genomes for each ST obtained from the National Center for Biotechnology Information (NCBI) reference sequence (RefSeq) database (O'Leary et al., 2016) using Breseq v.0.35.4 (Deatherage & Barrick, 2014) with default parameters. The predicted mutations in the selected genes of the study isolates were verified by aligning their respective nucleotide and amino acid sequences with the reference sequences using clustal omega (Sievers & Higgins, 2014). The impact of amino acid substitutions (neutral or deleterious) on the biological function of proteins was predicted using the protein variation effect analyzer v.1.1.3 (PROVEAN) tool (Choi & Chan, 2015).

## 3 RESULTS AND DISCUSSION

### 3.1 Antibiotic susceptibility patterns of wastewater *E. coli*

To understand whether *E. coli* isolates from wastewater have unique or unusual antibiotic resistance patterns, we assessed 12 CR‐EC and 151 ESBL‐EC isolates for resistance against 19 common antibiotics (Online Supporting Information: Table S1; https://figshare.com/search?q=10.6084%2Fm9.figshare.25356127). Based on the observed patterns, we selected several isolates for genome analysis due to their resistance pattern relevant to medical treatment as described below. Here, we refer to antibiotics according to the categories “critically important”, “highly important”, and “important” based on the definition of the World Health Organization (WHO, 2019) (further details in Online Supporting Information; https://figshare.com/search?q=10.6084%2Fm9.figshare.25356127).

Among the resistance patterns observed, some CR‐EC isolates exhibited high‐level resistances against almost all antibiotics tested (Online Supporting Information: Table S1; https://figshare.com/search?q=10.6084%2Fm9.figshare.25356127), which is rare in Australia based on data from human clinical isolates (AURA, 2021; Fasugba et al., 2019; Hastak et al., 2020). We selected CR‐EC isolates G4, G5, G8, and G16, which were resistant to all the tested highest‐priority, critically important, and highly important antibiotics (Table 1) as well as almost all high‐priority, critically important, and important antibiotics with variable patterns (Table 1). These isolates can thus be considered extensively drug‐resistant (XDR) *E. coli*, which are extremely challenging to treat, resulting in prolonged hospitalization, increased treatment costs, and poor patient outcomes (Batalla‐Bocaling et al., 2021; Magiorakos et al., 2012).

**Table 1:** Antibiotic susceptibility pattern of the carbapenem‐resistant *Escherichia coli* (CR‐EC) and extended‐spectrum β‐lactamases‐producing *E. coli* (ESBL‐EC) isolated from wastewater and selected for genome analysis.

Isolates	Year of collection	Phenotypes	Antibiotic Classes																		
			β‐lactams							Penicillins with β‐lactamase inhibitors			Aminoglycosides			Fluoroquinolones		Dihydrofolate reductase inhibitors	Combinations	Nitrofuran derivatives	Phosphonic acidderivatives
			Aminopenicillin	Cephamycin	Cephalosporins				Carbapenem												
			Ampicillin	Cefoxitin	Cefazolin	Ceftazidime	Ceftriaxone	Cefepime	Meropenem	Amoxicillin‐clavulanic acid	Piperacillin‐tazobactam	Ticarcillin‐clavulanic acid	Gentamicin	Tobramycin	Amikacin	Ciprofloxacin	Norfloxacin	Trimethorprim	Trimethoprim‐sulfamethoxazole	Nitrofurantoin	Fosfomycin
G4	2017	CR‐EC	**R** (≥32^a^)	**R** (≥64)	**R** (≥64)	**R** (≥64)	**R** (≥64)	**R** (≥64)	**R** (≥16)	**R** (≥32)	**R** (≥128)	**R** (≥128)	**R** (≥16)	**R** (≥16)	**R** (≥64)	**R** (≥4)	**R** (≥16)	**R** (≥16)	**R** (≥320)	**I** (64)	**S** (27 mm^b^)
G5	2017	CR‐EC	**R** (≥32)	**R** (≥64)	**R** (≥64)	**R** (≥64)	**R** (≥64)	**R** (≥64)	**R** (≥16)	**R** (≥32)	**R** (≥128)	**R** (≥128)	**R** (≥16)	**R** (≥16)	**R** (≥64)	**R** (≥4)	**R** (≥16)	**R** (≥16)	**R** (≥320)	**I** (64)	**S** (27 mm)
G8	2018	CR‐EC	**R** (≥32)	**R** (≥64)	**R** (≥64)	**R** (≥64)	**R** (≥64)	**R** (≥64)	**R** (8)	**R** (≥32)	**R** (≥128)	**R** (≥128)	**R** (≥16)	**R** (≥16)	**R** (≥64)	**R** (≥4)	**R** (≥16)	**R** (≥16)	**R** (≥320)	**R** (128)	**S** (23 mm)
G16	2019	CR‐EC	**R** (≥32)	**R** (≥64)	**R** (≥64)	**R** (≥64)	**R** (≥64)	**R** (≥64)	**R** (≥16)	**R** (≥32)	**R** (≥128)	**R** (≥128)	**R** (≥16)	**R** (≥16)	**S** (4)	**R** (≥4)	**R** (≥16)	**R** (≥16)	**R** (≥320)	**I** (64)	**S** (26 mm)
G10	2018	ESBL‐EC	**R** (≥32)	**R** (32)	**R** (≥64)	**R** (16)	**R** (≥64)	**R** (8)	**S** (≤0.25)	**R** (≥32)	**I** (64)	**S** (16)	**R** (≥16)	**I** (8)	**S** (≤2)	**I** (0.5)	**S** (2)	**S** (≤0.5)	**S** (≤20)	**R** (128)	**S** (21 mm)
G11	2018	ESBL‐EC	**R** (≥32)	**R** (≥64)	**R** (≥64)	**R** (≥64)	**R** (≥64)	**R** (≥64)	**S** (≤0.25)	**R** (≥32)	**R** (≥128)	**R** (≥128)	**S** (≤1)	**S** (≤1)	**S** (≤2)	**R** (2)	**S** (4)	**S** (2)	**S** (≤20)	**R** (128)	**S** (21 mm)
G15	2019	ESBL‐EC	**R** (≥32)	**R** (32)	**R** (≥64)	**R** (8)	**R** (≥64)	**R** (32)	**S** (≤0.25)	**S** (8)	**S** (16)	**S** (16)	**R** (≥16)	**I** (8)	**S** (≤2)	**S** (≤0.25)	**S** (2)	**R** (≥16)	**R** (≥320)	**R** (256)	**I** (14 mm)
G17	2019	ESBL‐EC	**R** (≥32)	**R** (≥64)	**R** (≥64)	**R** (≥64)	**R** (≥64)	**R** (32)	**S** (≤0.25)	**R** (≥32)	**I** (64)	**R** (≥128)	**R** (≥16)	**S** (2)	**S** (≤2)	**R** (2)	**S** (4)	**R** (≥16)	**R** (≥320)	**R** (128)	**S** (28 mm)
**Categories of important antibiotics for human medicine (WHO**, 2019 **)**			Critically important (High priority)	Highly important	Highly important	Critically important (Highest priority)	Critically important (Highest priority)	Critically important (Highest priority)	Critically important (High priority)	Critically important (High priority)	Critically important (High priority)	Not available	Critically important (High priority)	Critically important (High priority)	Critically important (High priority)	Critically important (Highest priority)	Critically important (Highest priority)	Highly important	Highly important	Important	Critically important (High priority)

Abbreviations: I, intermediate; R, resistant; S, sensitive.

^a^ MIC (µg/mL).

^b^ Inhibition zone diameter (nearest whole millimeter [mm]).

Among the diverse resistance patterns observed for ESBL‐EC (Online Supporting Information: Table S1; https://figshare.com/search?q=10.6084%2Fm9.figshare.25356127), we chose four isolates exhibiting resistance against the highest number of critically important and highly important antibiotics (WHO, 2019) (Table 1). Specifically, ESBL‐EC isolates G10, G11, G15, and G17 were MDR as they were resistant to at least three classes of antibiotics, including all tested β‐lactam antibiotic classes except carbapenems and nitrofurantoin (Table 1) (Magiorakos et al., 2012). Variable resistant patterns were observed in these isolates against the rest of the tested antibiotics (Table 1). ESBL‐EC isolates that are resistant to all the commonly prescribed β‐lactam antibiotics may limit treatment options and may increase the use of last‐resort drugs, such as carbapenem, as first‐line treatment (Harris et al., 2015). Furthermore, the diverse resistance pattern of ESBL‐EC isolates against other tested critically important antibiotics is concerning, as the acquisition of resistance against these classes will further reduce therapeutic options.

### 3.2 Genome and phylogenetic analysis

The general genomic characteristics of the *E. coli* isolates from wastewater are given in Online Supporting Information: Table S2 (https://figshare.com/search?q=10.6084%2Fm9.figshare.25356127). Quality‐filtered reads were assembled into between 73 and 469 contigs per genome, with an N50 between 61 and 213 kilobase pairs (kbp). The approximate size of the assembled genomes ranges from 4.87 to 6.13 megabase pairs (Mbp), with GC content between 50.46% and 51.81% (Online Supporting Information: Table S2; https://figshare.com/search?q=10.6084%2Fm9.figshare.25356127). Annotation of the genomes predicted 4498 and 5943 coding sequences (CDS) (Online Supporting Information: Table S2; https://figshare.com/search?q=10.6084%2Fm9.figshare.25356127).

**Figure 1:**
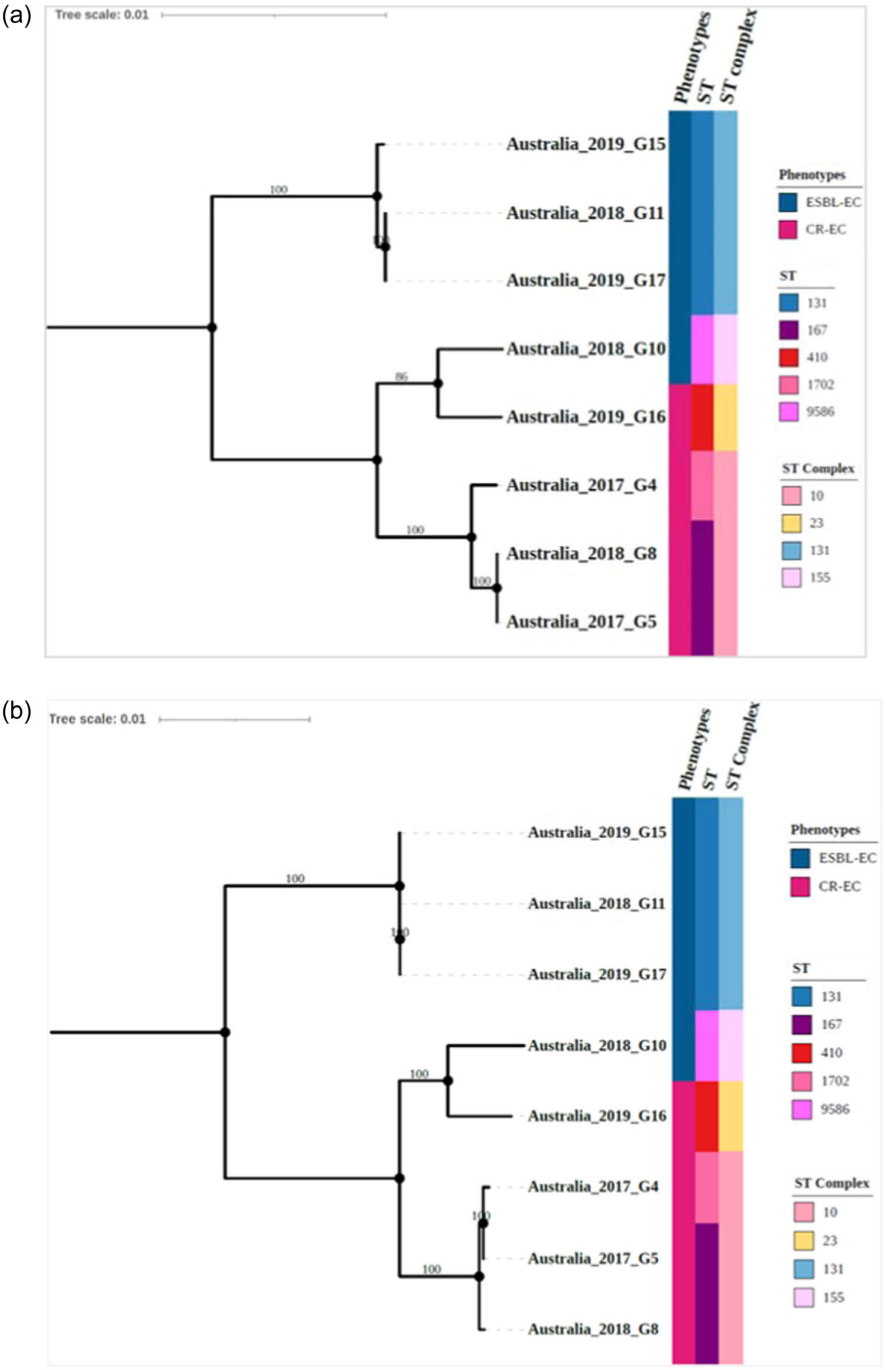
Maximum likelihood phylogenetic trees of *Escherichia coli* isolates from wastewater. (a) The tree was inferred from the alignment of concatenated MLST gene alleles (*adk–fumC–gyrB–icd–mdh–purA–recA*) extracted from the genome assembly applying the general time reversible (GTR)‐Gamma model with 1000 bootstraps in RAxML and is midpoint rooted. (b) The phylogeny was inferred from an alignment of concatenated core CDS (*n* = 3289) determined by Roary and RAxML with the GTR‐Gamma model and 1000 bootstrap iterations and is midpoint rooted. The tree nodes are labeled with the origin, year of isolation, and ID of the isolates. The colored strips indicate different phenotypes, sequence types (ST), and the respective ST complexes of the isolates.

Phylogenetic analysis of these wastewater isolates based on Achtman's MLST scheme (Wirth et al., 2006) revealed diverse STs (Figure 1a). Further resolution of the tree using 3,289 concatenated core CDS demonstrated concordance with the MLST‐based clustering (Figure 1b). The CR‐EC isolate G16 belonged to the internationally emerging high‐risk ST410 of the ST23 clonal complexes (Roer et al., 2018) (Figure 1). The CR‐EC isolates G5 and G8 belonged to the ST167 cluster and were closely related to the ST1702 G4 isolate (Figure 1). The CR‐EC isolates G4, G5, and G8 belonged to the ST10 clonal complex (Figure 1). ST167 was found to have emerged from ST10 clones and has frequently been reported worldwide and is commonly associated with carbapenem resistance in humans and other animals (Garcia‐Fernandez et al., 2020). The ESBL‐EC isolates G11, G15, and G17 were closely related and thus likely belonging to the globally disseminated, highly virulent ST131 (Mathers et al., 2015; Pitout & Finn, 2020) and its clonal complexes (Figure 1). The ESBL‐EC isolates G10 belonged to ST9586 (within the ST155 clonal complex) and is unique to Australia (Figure 1 and Online Supporting Information: Figure S8; https://figshare.com/search?q=10.6084%2Fm9.figshare.25356127).

Further core CDS‐based phylogenetic analysis with randomly selected global isolates showed that wastewater analysis detected strains mostly from human sources (G4, G5, G8, G16, G11, G15, and G17), but also strains (G10) that have previously been predominantly found in nonhuman sources (see Online Supporting Information for more details; https://figshare.com/search?q=10.6084%2Fm9.figshare.25356127). In addition, we found that wastewater testing can detect new strains (G10) that have previously not been found in the local Australian setting (Online Supporting Information; https://figshare.com/search?q=10.6084%2Fm9.figshare.25356127).

### 3.3 Genetic determinants of antibiotic resistance

As the previous section has highlighted that the wastewater isolates possess several unique antibiotic resistance profiles, we next examined the molecular mechanisms underpinning resistance. ResFinder predicted multiple antibiotic resistance genes against clinically significant antibiotic classes from the genomes of the XDR CR‐EC and MDR ESBL‐EC isolates (Table 2).

**Table 2:** Genetic characteristics of the carbapenem‐resistant *Escherichia coli* (CR‐EC) and extended‐spectrum β‐lactamases‐producing *E. coli* (ESBL‐EC) isolates.

Isolates	MLST types	Phenotypes	Antibiotic resistance pattern	Antibiotic resistance genes	Virulence‐associated genes	Plasmid replicons
G4	1702	CR‐EC	AMP, FOX, CFZ, CAZ, CRO, FEP, MEM, AMC, TZP, TIM, GEN, TOB, AMK, CIP, NOR TMP, SXT	*bla*_NDM‐5_, *bla*_CMY‐42_, *bla*_GES‐5_, *bla*_TEM‐1B_, *rmtB*, *aac(6′)‐Ib3*, *aac(6′)‐Ib‐cr*, *qnrS2*, *gyrA* (S83L, D87N), *parC* (S80I, P578L), *parE* (S458A), *dfrA12, sul1*	**Adhesin:** *fimH, hra*; **siderophore/iron uptake:** *fyuA, irp2*; **protectins/serum resistance:** *iss, traT*; **other:** *capU, terC*	Col (pHAD28), ColRNAI, IncFIA, IncFII, IncI (Gamma), IncQ2
G5	167	CR‐EC	AMP, FOX, CFZ, CAZ, CRO, FEP, MEM, AMC, TZP, TIM, GEN, TOB, AMK, CIP, NOR, TMP, SXT	*bla*_NDM‐5_, *bla*_IMP‐4_, *bla*_TEM‐1B_, *aac(6′)‐Ib3*, *aac(3)‐IId*, *aac(6′)‐Ib‐cr*, *qnrA1, gyrA* (S83L, D87N), *parC* (S80I, P578L), *parE* (S458A), *dfrA12, sul1*	**Adhesin:** *fimH, hra*; **siderophore/iron uptake:** *fyuA, irp2*; **protectins/serum resistance:** *iss, traT*; **other:** *capU, terC*	Col156, Col (pHAD28), Col440I, Col440II, ColRNAI, FII (pBK30683), IncFIA, IncFIB (AP001918), IncFIB (K), IncFII, IncFII (K), IncM2, IncR
G8	167	CR‐EC	AMP, FOX, CFZ, CAZ, CRO, FEP, MEM, AMC, TZP, TIM, GEN, TOB, AMK, CIP, NOR, TMP, SXT, NIT	*bla*_NDM‐5_, *bla*_CMY‐145_, *bla*_TEM‐1B_, *rmtB, gyrA* (S83L, D87N), *parC* (S80I, P578L), *parE* (S458A), *dfrA12*, *sul1*, *nfsA*, *nfsB*	**Adhesin:** *hra*; **siderophore/iron uptake:** *iucC, iutA*; **protectins/serum resistance:** *iss, traT*; **other:** *capU, gad, terC*	IncF1A, IncFIB (AP001918), IncFII, IncI (Gamma)
G16	410	CR‐EC	AMP, FOX, CFZ, CAZ, CRO, FEP, MEM, AMC, TZP, TIM, GEN, TOB, CIP, NOR, TMP, SXT	*bla*_NDM‐5_, *bla*_OXA‐1_, *bla*_CMY‐2_, *bla*_TEM‐1B_, *bla*_CTX‐M‐15,_ *aac(3)‐IId, aac(6′)‐Ib‐cr*, *gyrA* (S83L, D87N), *parC* (S80I), *parE (S458A), dfrA12*, *dfrA17, sul1*, *sul2*	**Adhesin:** *fimH, hra, ipfA*; **enterotoxin:** *astA*; **other:** *gad, terC*	Col (BS512), IncFIA, IncFIB (AP001918), IncFII (pAMA1167‐NDM‐5), IncQ1
G10	9586	ESBL‐EC	AMP, FOX, CFZ, CAZ, CRO, FEP, AMC, GEN, NIT	*bla*_CTX‐M‐55_, *ampC*‐promoter (−42, C‐ > T), *aac(3)‐IId*, *qnrS1*, *nfsA*, *nfsB*	**Adhesins:** *ipfA*; **siderophore/iron uptake:** *fyuA, irp2, iucC, iutA, sitA*; **protectins/serum resistance:** *iss*; *ompT, traT* **; pore‐forming toxin:** *hlyF*; **secretion system:** *estC*; **other:** *gad, terC*	Col440II, Col (pHAD28), ColpVC, IncFIA, IncFIB (AP001918), IncFII
G11	131	ESBL‐EC	AMP, FOX, CFZ, CAZ, CRO, FEP, AMC, TZP, TIM, CIP, NIT	*bla*_CMY‐2_, *bla*_TEM‐1C_, *bla*_CTX‐M‐27_, *gyrA* (S83L, A828S), *gyrB* (A618T), *parC* (A117E, D475E, T718A), *parE* (V136I, I529L), *nfsA, nfsB*	**Adhesins:** *fimH, iha, papA_F43*; **siderophore/iron uptake:** *chuA, fyuA, irp2, iucC, iutA*; **protectins/serum resistance:** *kpsE, kpsMII_K5, ompT, traT*; **enterotoxin:** *senB*; **fimbrial protein:** *ycfV*; **toxin:** *sat*; **bacteriocin:** *usp*; **other:** *gad, terC*	Col156, IncFIB (AP001918), IncFII (29), IncFII (pRSB107), IncI (Gamma)
G15	131	ESBL‐EC	AMP, FOX, CFZ, CAZ, CRO, FEP, GEN, TMP, SXT, NIT	*bla*_TEM‐1B_, *bla*_CTX‐M‐27_, *aac(3)‐IId*, *gyrA* (S83L, A828S), *gyrB* (A618T), *parC* (D475E, T718A), *parE* (V136I, I529L), *dfrA17, sul1, sul2, nfsA, nfsB*	**Adhesins:** *fimH, iha, papA_F43, papC*; **siderophore/iron uptake:** *chuA, fyuA, irp2, iucC, iutA, sitA*; **protectins/serum resistance:** *kpsE, kpsMII_K5, ompT, traT*; **enterotoxin:** *senB*; **fimbrial protein:** *ycfV*; **toxin:** *cnf1, sat*; **bacteriocin:** *usp*; **other:** *gad, terC*	Col156, IncFIB (AP001918), IncFII (29)
G17	131	ESBL‐EC	AMP, FOX, CFZ, CAZ, CRO, FEP, AMC, TIM, GEN, CIP, TMP, SXT, NIT	*bla*_CMY‐2_, *bla*_CMY‐58_, *bla*_TEM‐1C_, *bla*_CTX‐M‐27_, *aac(3)‐IId*, *gyrA* (S83L, A828S), *gyrB* (A618T), *parC* (A117E, D475E, T718A), *parE* (V136I, I529L), *dfrA17, sul1, sul2, nfsA, nfsB*	**Adhesins:** *fimH, iha, papA_F43*; **siderophore/iron uptake:** *chuA, fyuA, irp2*; **protectins/serum resistance:** *kpsE, kpsMII_K5, traT, ompT*; **enterotoxin:** *senB*; **fimbrial protein:** *ycfV*; **bacteriocin:** *usp*; **other:** *gad, terC*	Col156, IncFIB (AP001918), IncFII (29), IncFII (pRSB107), IncI (Gamma)

Abbreviations: AMC, Amoxicillin‐clavulanic acid; AMK, Amikacin; AMP, Ampicillin; CAZ, Ceftazidime; CFZ, Cefazolin; CIP, Ciprofloxacin; CRO, Ceftriaxone; FEP, Cefepime; FOX, Cefoxitin; GEN, Gentamicin; MEM, Meropenem; NIT, Nitrofurantoin; NOR, Norfloxacin; SXT, Trimethoprim‐sulfamethoxazole; TIM, Ticarcillin‐clavulanic acid; TMP, Trimethoprim; TOB, Tobramycin; TZP, Piperacillin‐tazobactam.

### 3.4 Resistance to β‐lactams could be mediated by diverse genes encoding ESBLs and carbapenemases

Among the different β‐lactamases produced by *E. coli*, ESBLs, AmpC β‐lactamases, and carbapenemases remain the most significant from a clinical point of view (Meini et al., 2019; Peirano & Pitout, 2019). Carbapenem resistance in the XDR CR‐EC isolates G4 was mediated mainly by the co‐carriage of genes for the NDM‐5‐ and GES‐5‐type carbapenemases (Table 2), the co‐occurrence of NDM‐5 and IMP‐4 in isolate G5 (Table 2) and the carriage of NDM‐5 in isolates G8 and G16, which is consistent with their resistance to all tested β‐lactam antibiotics and β‐lactam inhibitors (Table 2) (Boyd et al., 2020). Furthermore, several deleterious mutations were observed in the *ompC* gene of CR‐EC isolates (Online Supporting Information: Table S3; https://figshare.com/search?q=10.6084%2Fm9.figshare.25356127) that could confer additional nonspecific carbapenem resistance when combined with ESBL and/or high levels of AmpC production, as previously reported (Liu et al., 2012). These mutations are further discussed in the Online Supporting Information (https://figshare.com/search?q=10.6084%2Fm9.figshare.25356127).

Resistance of the G10, G11, G15, and G17 isolates to ampicillin and cephalosporins could be explained by the presence of a gene for a CTX‐M‐type ESBL (Table 2) (Bush & Bradford, 2020; Paterson & Bonomo, 2005). In isolates G11 and G17, resistance to cefoxitin, amoxicillin‐clavulanic acid, piperacillin‐tazobactam, and ticarcillin‐clavulanic acid could be attributed to the carriage of genes for CMY‐2‐type AmpC β‐lactamases (Table 2) (Jacoby, 2009; Meini et al., 2019). In isolate G10, potential upregulating mutations in the promoter region of the *ampC* gene (position ‐42, C ‐> T mutation) might explain resistance to cefoxitin (Caroff et al., 1999; Peter‐Getzlaff et al., 2011) and amoxicillin‐clavulanic acid (Table 2). Although isolate G15 was phenotypically detected as being cefoxitin resistant, no previously identified mutations that upregulated the *ampC* gene (Caroff et al., 1999; Peter‐Getzlaff et al., 2011) and CMY‐type AmpC β‐lactamases were observed, indicating an unknown mechanism of resistance to cefoxitin. In addition, several deleterious mutations were predicted for OmpC of the carbapenem‐susceptible ESBL‐EC isolates (Online Supporting Information: Table S3; https://figshare.com/search?q=10.6084%2Fm9.figshare.25356127). However, these mutations may not be sufficient to confer a clinically relevant level of carbapenem resistance in these isolates, as discussed in the Online Supporting Information (https://figshare.com/search?q=10.6084%2Fm9.figshare.25356127).

### 3.5 Aminoglycoside resistance could be attributed to genes encoding various aminoglycoside‐modifying enzymes

Resistances to clinically significant aminoglycosides such as gentamicin, tobramycin, and amikacin in this study's isolates were likely mediated by various aminoglycoside acetyltransferases and 16S rRNA methyltransferases (RMTase) encoding genes (Table 2). Amikacin resistance in CR‐EC isolates G4 and G8 could be explained by the carriage of a 16S RMTases encoding gene (i.e., *rmtB*) (Table 2), which also would render these isolates resistant to tobramycin and gentamicin, as previously documented (Wachino et al., 2020). Isolate G4 also carried an *aac(6′)‐Ib3* gene encoding for the aminoglycoside 6′‐N‐acetyltransferase type Ib3 and an *aac(6′)‐Ib‐cr* gene encoding the aminoglycoside 6′‐N‐acetyltransferase type Ib‐cr, capable of conferring resistance to tobramycin and amikacin (Ramirez et al., 2013; Ramirez & Tolmasky, 2010; Wachino et al., 2020). Likewise, the occurrence of the *aac(6′)‐Ib3* and *aac(6′)‐Ib‐cr* genes in the CR‐EC isolate G5 likely conferred resistance to amikacin and tobramycin, and the carriage of the *aac(3)‐IId* gene encoding for the aminoglycoside 3‐N‐acetyltransferase type IId can explain gentamicin resistance (Table 2). In CR‐EC isolate G16, the presence of *aac(3)‐IId* and *aac(6′)‐Ib‐cr* genes could explain the resistances to gentamicin and tobramycin, respectively (Table 2) (Ramirez et al., 2013; Ramirez & Tolmasky, 2010). Interestingly, despite the presence of an *aac(6′)‐Ib‐cr* gene, isolate G16 was phenotypically detected as amikacin sensitive (Tables 1 and 2). The promoter region of the *aac(6′)‐Ib‐cr* gene was found to be unchanged, and no deleterious mutations were found in the gene. Therefore, the reason for the amikacin susceptibility of isolate G16 remains unclear. ESBL‐EC isolates G10, G15, and G17 were resistant only to gentamicin, which was correlated with the presence of *aac(3)‐IId* genes (Table 2).

### 3.6 Fluoroquinolone resistance is mainly correlated with mutations in the genes encoding the antibiotic's target enzymes

Resistances to ciprofloxacin and norfloxacin in the CR‐EC isolates G4, G5, G8, and G16 could be mainly attributed to mutations in the quinolone resistance‐determining region (QRDR) of the genes for the DNA gyrase GyrA and the topoisomerase IV subunit ParC, and, outside the QRDR, for the topoisomerase IV subunit ParE proteins (Table 2). For instance, isolates G4, G5, G8, and G16 have previously reported mutations in the genes *gyrA* and *parCE* that confer resistance (Table 2) (Hooper & Jacoby, 2016; Ruiz, 2003). A novel deleterious mutation in *parC* located outside the QRDR region was also found in isolates G4, G5, and G8 (Table 2).

Ciprofloxacin resistance in ESBL‐EC isolates G11 and G17 was likely mediated by mutations in the *gyrA* and *parC* genes, as previously documented (Table 2) (Hooper & Jacoby, 2016; Ruiz, 2003). These isolates also had neutral mutations outside the QRDR of *gyrAB* and *parCE*, and none had PMQR genes (Table 2). Interestingly, despite carrying these resistant‐conferring and neutral mutations, isolates G11 and G17 were phenotypically identified as sensitive to norfloxacin (Table 2). The mechanism for this genotype‐phenotype discrepancy remains unclear. As isolate G10 had wild‐type gene sequences for *gyrAB* and *parCE*, intermediate susceptibility to ciprofloxacin can be explained by the carriage of a *qnrS1* gene, which confers low‐level resistance (Jacoby et al., 2014). Although isolate G15 had a resistance mutation in *gyrA*, it was sensitive to ciprofloxacin and norfloxacin (Tables 1 and 2). In addition, this isolate did not have deleterious mutations in the *gyrB* and *parCE* genes and lacked PMQR genes (Table 2). Therefore, the mutation in *gyrA* alone was not sufficient to confer a clinically relevant level of fluoroquinolone resistance in isolate G15, as reported earlier (Morgan‐Linnell & Zechiedrich, 2007).

### 3.7 Resistance to trimethoprim and sulfamethoxazole is associated with genes encoding antibiotic‐resistant variants of the chromosomal target enzymes

Resistance to trimethoprim in the CR‐EC isolates G4, G5, G8, and G16 can be explained by the presence of the *dfrA12* gene encoding for a resistant variant of the antibiotic's target enzyme dihydrofolate reductase (Table 2), as reported previously (Sköld, 2001). Isolate G4, G5, G8, and G16 also harbored *dfrA12* and *sul1* genes (encoding a dihydropteroate synthase) that might confer resistance to the trimethoprim‐sulfamethoxazole combination, as per previous studies (Eliopoulos & Huovinen, 2001; Ho et al., 2009). The G16 isolate was found to have additional *dfrA17* and *sul2* genes (Table 2). Trimethoprim resistance in ESBL‐EC isolates G15 and G17 was likely mediated by the *dfrA17* gene, and trimethoprim‐sulfamethoxazole resistance could be conferred by the co‐occurrence of the *dfrA17* and *sul1, sul2* genes (Table 2).

### 3.8 Nitrofurantoin resistance is correlated with novel mutations in the nitroreductase encoding genes and likely unknown mechanisms

As none of the CR‐EC and ESBL‐EC isolates had *oqxAB*‐type genes encoded for multidrug efflux pump and resistance‐conferring mutations in the *ribE* gene, nitrofurantoin resistance is likely caused by mutations in the *nfsA* and *nfsB* genes that encode for the oxygen‐insensitive nitroreductases (Ho et al., 2016; Sandegren et al., 2008; Vervoort et al., 2014; Zhang et al., 2018). Comparison with the wild‐type *E. coli* J53 strain (GenBank accession number, AICK00000000.1) revealed several unique or previously reported mutations in the *nfsA* and *nfsB* genes of the eight isolates. For instance, a unique nine‐nucleotide (227–235) deletion, resulting in the loss of three amino acids (FWV, positions 76–78), was observed in the NfsA of the nitrofurantoin‐intermediate G4, G5, and resistant G8 CR‐EC isolates. PROVEAN analysis predicts that this deletion will have a deleterious effect on the enzymatic function of NfsA. The *nfsB* genes of these isolates were found to be unmutated, and unlike previous studies, no other previously reported mutations were found in the *nfsA* gene (Sandegren et al., 2008; Zhang et al., 2018). Moreover, the promoter region of the *nfsA* gene was found to be unchanged in these isolates. Therefore, some unknown mechanisms may cause elevated MIC in isolate G8 (128 μg/mL) compared to intermediate G4 and G5 isolates (64 μg/mL) (Table 1). A novel deleterious mutation (R203H) at the active site of NfsA in CR‐EC isolate G16 might cause intermediate susceptibility, as the substitutions (G66D, M75I, and V93A) found in NfsB were reported to be neutral (Sandegren et al., 2008; Zhang et al., 2018).

In the nitrofurantoin‐resistant ESBL‐EC isolate G10, a novel large deletion of 138 amino acid residues (del M‐A positions 1–138) was found in the N‐terminal region of NfsA containing two of the three nucleotide‐phosphate binding regions (11–15, and 128–131 aa) and FMN binding sites (39 and 67). NfsA reduces nitrofurantoin antibiotics using NADPH, while FMN serves as a cofactor that mediates electron transfer to various electron acceptors from NADPH (Kobori et al., 2001). As no other deleterious mutations were found in the *nfsA* and *nfsB* genes, the deletion of this functionally important region of NfsA may cause resistance in isolate G10. Nitrofurantoin resistance in ESBL‐EC isolates G11 and G17 may be mediated by identical and novel deleterious single nucleotide polymorphisms (SNPs) in *nfsA* (P3G, T4P, and I5S) and *nfsB* (G153D, in one of the four nucleotide‐phosphate binding regions) genes. An unknown mechanism may drive the nitrofurantoin resistance in G15, as this isolate only has previously described neutral mutations in the *nfsA* (I117T, K141E, and G187D) and *nfsB* (G66D and V93A) genes (Vervoort et al., 2014; Zhang et al., 2018).

### 3.9 Deleterious mutations in the regulatory genes of MDR efflux pumps may contribute to resistance

Various insertion‐deletions (INDELs) and SNPs were detected for the CR‐EC isolates G4, G5, and G8 in the *acrR* gene, which encodes for a protein belonging to the TetR‐family transcriptional regulators involved in the regulation of the multidrug efflux pump (Colclough et al., 2019). Some deleterious SNPs were found in the N‐terminal conserved region (10–70 aa) of AcrR (A2G, R3T, T5N, K6Q, Q7T, A9S, E11R, T12N, R13A, Q14P, H15T, I16H, L17P, D18R, A20G, L21S, and R22T), which likely has some adverse effects on the repression of the multidrug efflux in XDR CR‐EC isolates G4, G5, and G8.

Overall, the analysis of resistance mechanisms showed that most of the resistance against β‐lactams, aminoglycosides, fluoroquinolones, trimethoprim, and sulfamethoxazole was caused by diverse combinations of previously defined clinically relevant resistant determinants. However, novel resistance mechanisms against nitrofurantoin antibiotics were predicted in the wastewater isolates based on unique deleterious mutations in *nfsAB*. In addition, no known genetic bases of resistance against some antibiotics such as cephamycin (i.e., cefoxitin) were found, indicating the existence of a novel mechanism. The overexpressed MDR efflux due to deleterious mutations in regulatory genes might constitute novel multidrug resistance mechanisms in isolates lacking other resistance determinants.

### 3.10 Virulence gene content classified some isolates into extraintestinal pathotypes

We next sought to investigate whether the MDR and XDR wastewater isolates also have the genomic potential to be pathogenic to humans and animals. VirulenceFinder predicted multiple virulence genes from the genomes of the CR‐EC and ESBL‐EC isolates (Table 2).

Specifically, ESBL‐EC isolates G11, G15, and G17 belonging to ST131 had multiple virulence factors in variable combinations, including adhesins, siderophore/iron uptake system, protectins/serum resistance, enterotoxin, and toxins (Table 2). These isolates also had putative virulence factors such as bacteriocins, and fimbrial proteins (Table 2). Although the isolates G11, G15, and G17 carried a considerable number of extra‐intestinal, infection‐related genes shared between different pathotypes (Riley, 2014; Sarowska et al., 2019), the presence of the uropathogenic *E. coli* (UPEC) predictor genes *chuA*, *fyuA* (siderophore/iron uptake), and *ycfV* (fimbrial protein) would classify these isolates as UPEC (Spurbeck et al., 2012). The ESBL‐EC isolate G10 belonging to ST9586 also harbored several virulence factors such as siderophores, protectins, adhesin, pore‐forming toxins, and secretion systems (Table 2). The G10 isolate could be classified as avian pathogenic *E. coli* (APEC)‐like owing to the presence of the *hlyF* (pore‐forming toxin), *iss, ompT* (protectins), and *iutA* (siderophores) predictor genes (Johnson et al., 2008).

CR‐EC isolates G4, G5, and G8 belonging to ST167 had several virulence factors such as adhesins, siderophores, and protectins (Table 2). Although G4 and G5 isolates had the APEC‐related gene *iss* and the UPEC‐associated gene *fyuA*, and G8 carried the APEC‐related genes *iss* and *iutA*, these isolates could not be assigned to respective pathotypes due to the absence of a required number of other predictor genes (Johnson et al., 2008; Spurbeck et al., 2012). The CR‐EC isolate G16 (ST410 type) harbored several genes encoding adhesins and enteroaggregative *E. coli* associated enterotoxin (*astA*) (Table 2). However, the isolate G16 could not be classified as a specific diarrheagenic pathotype due to the lack of other genetic markers (Fujioka et al., 2013).

Pathotyping based on the virulence gene content thus indicated that all MDR ESBL‐EC isolates from wastewater isolates belong to known extra‐intestinal pathotypes of humans and animals. However, despite carrying various extra‐intestinal and intestinal pathotype‐related genes, none of the XDR CR‐EC isolates were classified into known pathotypes due to the lack of the required number of virulence markers. The lack of a sufficient number of virulence markers might also indicate that these *E. coli* isolates may not be able to cause symptomatic infections and, therefore, might not be sampled in diagnostic settings.

### 3.11 The potential of wastewater isolates for the acquisition or transfer of antibiotic resistance and virulence genes

We next investigated whether wastewater isolates have acquired or could transfer these genes. PlasmidFinder identified that the XDR CR‐EC isolate G4, G5, G8, and G16 harbored several colicinogenic (Col)‐ and Inc‐type plasmid replicons (Table 2). The contig harboring the IncQ2 replicon in isolate G4 also had the *qnrS2* gene, suggesting plasmid‐mediated acquisition (Hayer et al., 2020; Wen et al., 2016) (Online Supporting Information: Table S4; https://figshare.com/search?q=10.6084%2Fm9.figshare.25356127). Likewise in isolates G5 and G16, the acquisition of the *bla*_TEM‐1B_ and *sul2* genes may be associated with the IncM2 and IncQ1 plasmid, respectively (Online Supporting Information: Table S4;https://figshare.com/search?q=10.6084%2Fm9.figshare.25356127), as reported recently (Dor et al., 2020; Rozwandowicz et al., 2018).

The MDR ESBL‐EC isolates G10, G11, G15, and G17 harbored various Col‐ and Inc‐type plasmid replicons in various combinations (Table 2). In isolate G10, the acquisition of the genes *hylF* (toxin) and *ompT* (serum resistance) may be associated with the IncF‐type plasmid (Online Supporting Information: Table S4; https://figshare.com/search?q=10.6084%2Fm9.figshare.25356127), according to previous reports (Mellata et al., 2009; Olsen et al., 2012). The carriage of the *senB* (enterotoxin) gene in the G11, G15, and G17 isolates might be related to the Col156 plasmid (Online Supporting Information: Table S4; https://figshare.com/search?q=10.6084%2Fm9.figshare.25356127). In isolate G15, the carriage of *bla*_TEM‐1B_ was possibly associated with the IncF‐type plasmid (Online Supporting Information: Table S4;https://figshare.com/search?q=10.6084%2Fm9.figshare.25356127), as previously reported (Kim et al., 2011).

Overall, we found evidence for the plasmid‐mediated acquisition of several clinically relevant resistance and virulence genes in the XDR and MDR wastewater isolates. Identification of plasmid‐borne resistance genes in some of the XDR CR‐EC isolates of unknown pathotypes indicates that these isolates could serve as underexplored reservoirs of transferrable resistance genes.

### 3.12 Impact of mutations on bacterial survival and motility

Breseq and PROVEAN analyses identified several mutations with predicted functional impacts on the survival and motility of the wastewater isolates. Deletions and several nonsynonymous SNPs were found in the *nfrA* gene, encoding an outer membrane receptor for the N4 bacteriophage adsorption, in isolates G4, G11, and G16 (Kiino & Rothman‐Denes, 1989) (Online Supporting Information: Table S5; https://figshare.com/search?q=10.6084%2Fm9.figshare.25356127). These include the loss of the signal peptides of NfrA in the G11 isolate that may prevent its translocation to the outer membrane (Kiino & Rothman‐Denes, 1989) and thus impair N4 bacteriophage adsorption. Similarly, mutations in isolate G16 fall in one of the three TPR domains (i.e., TPR 2), used by phage N4 for irreversible interaction with NfrA (McPartland & Rothman‐Denes, 2009). Therefore, these mutations likely provide a survival advantage by preventing phage‐mediated lysis.

Frameshift mutations due to INDELs and various nonsynonymous SNPs were identified in the *cirA* gene of isolates G4, G5, G8, and G10, whose product serves as an outer membrane receptor for colicins IA and IB (Buchanan et al., 2007). These deletions that remove the colicin binding region and most of the Cir receptor (Buchanan et al., 2007), and nonsynonymous SNPs in the plug domain of the receptor might impair the translocation of colicin inside the cell (Online Supporting Information: Table S5; https://figshare.com/search?q=10.6084%2Fm9.figshare.25356127). Therefore, these mutations may offer a survival advantage by preventing colicin‐mediated killing in competitive niches.

Various structural variations were also observed in genes associated with bacterial motility, such as *flhA* and *fliI* in isolates G4, G5, and G8, and *fliP* in isolate G10. FlhA is one of the six integral membrane proteins of the flagellar export apparatus required to form the rod structure (Minamino & Macnab, 1999). A large deletion in isolates G4, G5, and G8 (Online Supporting Information: Table S5; https://figshare.com/search?q=10.6084%2Fm9.figshare.25356127) was predicted to impair the export of flagellar apparatus proteins, resulting in an adverse effect on motility (McMurry et al., 2004).

Also related to motility is FliI, which is an ATPase that hydrolyzes ATP to provide energy for the translocations of flagellar export substrates and is responsible for flagellar assembly across the cytoplasmic membrane (Fan & Macnab, 1996). A large deletion in isolates G4, G5, and G8 that removes the region with ATPase activity required to translocate the flagellar export apparatus across the membrane was predicted to be deleterious (Online Supporting Information: Table S5; https://figshare.com/search?q=10.6084%2Fm9.figshare.25356127). Therefore, this mutation is likely to have an adverse effect on motility of these isolates.

A frameshift mutation due to deletion that removes part of the conserved pore‐forming transmembrane segment 4 (TM4) and an SNP were observed in the *fliP* gene of isolate G10 (Online Supporting Information: Table S5; https://figshare.com/search?q=10.6084%2Fm9.figshare.25356127). FliP is one of the essential membrane components of the flagellar export apparatus required for the formation of the protein‐conducting pore of the flagellar secretion apparatus (Ward et al., 2018). These mutations may impair the transport of flagellar assembly proteins (Erhardt et al., 2017) and, therefore, are likely to have an adverse effect on motility.

Overall, we found that some CR‐EC and ESBL‐EC isolates from wastewater evolved not only to become resistant to multiple antibiotics but also likely to prevent phage predation and colicin‐mediated killing that may enable them to survive and proliferate in diverse competitive niches, which is concerning. In addition, we observed mutations that are predicted to impact motility in some CR‐EC and ESBL‐EC isolates. This might adversely influence their ability to successfully colonize humans and animals host to cause infections (Josenhans & Suerbaum, 2002), however, this requires further investigation. This study is limited as it focused only on a few *E. coli* isolates with unusual resistance patterns, which do not represent the overall antibiotic resistance pattern of all wastewater isolates, which warrants future studies.

## 4 CONCLUSION

In this study, we found that the selected XDR CR‐EC and MDR ESBL‐EC isolates from wastewater exhibit resistance against critically important antibiotics, are phylogenetically related to pandemic high‐risk human‐associated clones (ST131, ST167, ST410), and to emerging human‐ and nonhuman‐associated clones (ST1702, ST9586) and have unique and novel genetic features related to resistance mechanisms, virulence and environmental fitness. Wastewater‐based surveillance thus has a clear potential to monitor resistance beyond clinical settings and can complement healthcare infection surveillance to inform infection control and prevention strategies to effectively track and control the emergence and spread of antibiotic resistance.

## AUTHOR CONTRIBUTIONS

**Zillur Rahman**: Conceptualization (equal); formal analysis (lead); investigation (equal); methodology (equal); software (lead); visualization (lead); writing—original draft (lead); writing—review & editing (equal). **Mary‐Louise McLaws**: Conceptualization (equal); investigation (equal); project administration (equal); resources (equal); supervision (equal). **Torsten Thomas**: Conceptualization (equal); formal analysis (equal); investigation (equal); methodology (equal); resources (lead); supervision (lead); visualization (equal); writing—original draft (equal); writing—review & editing (equal).

## CONFLICT OF INTEREST STATEMENT

None declared

## ETHICS STATEMENT

None required.

## Supporting information

 

## Data Availability

The sequence data from this study are available in the GenBank database under accession numbers JAQAHI000000000, JAQAHJ000000000, JAQAHM000000000, JAQAHO000000000, JAQAHP000000000, JAQAHT000000000, JAQAHU000000000, JAQAHV00000000 (https://www.ncbi.nlm.nih.gov/bioproject/916637).
