# Supplementary material for: Genomic characterization of extended‐spectrum beta‐lactamase‐producing and carbapenem‐resistant Escherichia coli from urban wastewater in Australia
Source: Microbiologyopen. 2024 Mar 15;13(2):e1403. doi: 10.1002/mbo3.1403 (PMC10941799; doi:10.1002/mbo3.1403)
Supplement: Supplementary file 1 [file MBO3-13-e1403-s001.docx]

**Appendix 2**

**Table S1.** Antibiotic susceptibility pattern of the carbapenem-resistant *E. coli* (CR-EC) (n=12) and extended-spectrum β-lactamases-producing *E. coli* (ESBL-EC) (n=151) isolated from wastewater-based surveillance in Sydney at six-time points between 2017 and 2019. Isolates selected for whole-genome sequencing are in bold and highlighted in orange for resistant (R), blue for intermediate susceptible (I), and green for susceptible isolates (S).

| **Sequenced Genome Number** | **Month and year of isolation** | **Wastewater catchments** | **Phenotypes** | **Antibiotic Classes** | | | | | | | | | | | | | | | | | | |
| --- | --- | --- | --- | --- | --- | --- | --- | --- | --- | --- | --- | --- | --- | --- | --- | --- | --- | --- | --- | --- | --- | --- |
|  |  |  |  | **β-lactams** | | | | | | | **Penicillins with β-lactamase inhibitors** | | | **Aminoglycosides** | | | **Fluoroquinolones** | | **Dihydrofolate reductase inhibitors** | **Combinations** | **Nitrofuran derivatives** | **Phosphonic acid derivatives** |
|  |  |  |  | **Aminopenicillin** | **Cephamycin** | **Cephalosporins** | | | | **Carbapenem** |  |  |  |  |  |  |  |  |  |  |  |  |
|  |  |  |  | Ampicillin | Cefoxitin | Cefazolin | Ceftazidime | Ceftriaxone | Cefepime | Meropenem | Amoxicillin-clavulanic acid | Piperacillin-tazobactam | Ticarcillin-clavulanic acid | Gentamicin | Tobramycin | Amikacin | Ciprofloxacin | Norfloxacin | Trimethoprim | Trimethoprim-sulfamethoxazole | Nitrofurantoin | Fosfomycin |
| **G4** | **July 2017** | **West Hornsby** | **CR-EC** | **R** | **R** | **R** | **R** | **R** | **R** | **R** | **R** | **R** | **R** | **R** | **R** | **R** | **R** | **R** | **R** | **R** | **I** | **S** |
| **G5** | **July 2017** | **Riverstone** | **CR-EC** | **R** | **R** | **R** | **R** | **R** | **R** | **R** | **R** | **R** | **R** | **R** | **R** | **R** | **R** | **R** | **R** | **R** | **I** | **S** |
| **G8** | **May 2018** | **Bondi** | **CR-EC** | **R** | **R** | **R** | **R** | **R** | **R** | **R** | **R** | **R** | **R** | **R** | **R** | **R** | **R** | **R** | **R** | **R** | **R** | **S** |
| **G16** | **February 2019** | **Malabar** | **CR-EC** | **R** | **R** | **R** | **R** | **R** | **R** | **R** | **R** | **R** | **R** | **R** | **R** | **S** | **R** | **R** | **R** | **R** | **I** | **S** |
|  | February 2018 | Cronulla | CR-EC | R | R | R | S | R | S | R | R | R | S | S | S | S | R | R | R | R | S | S |
|  | May 2018 | Quakers Hill | CR-EC | R | R | R | R | R | R | R | R | R | S | S | I | S | R | R | R | R | S | S |
|  | September 2018 | Bondi | CR-EC | R | R | R | R | R | R | R | R | R | R | S | S | S | S | S | R | R | S | S |
|  | September 2018 | Rouse Hill | CR-EC | R | S | R | R | R | R | S | S | S | S | S | S | S | S | S | R | R | S | S |
|  | February 2019 | Riverstone | CR-EC | R | R | R | R | R | R | R | R | R | R | S | R | S | R | R | R | R | I | S |
|  | June 2019 | Riverstone | CR-EC | R | R | R | R | R | R | R | R | R | R | S | R | S | R | R | R | R | I | S |
|  | June 2019 | West Camden | CR-EC | R | R | R | R | R | R | R | R | R | R | R | I | S | R | R | S | S | S | S |
|  | June 2019 | Warriewood | CR-EC | R | R | R | R | R | R | S | R | R | R | R | S | S | R | S | R | R | R | S |
| **G10** | **September 2018** | **Warriewood** | **ESBL-EC** | **R** | **R** | **R** | **R** | **R** | **R** | **S** | **R** | **I** | **S** | **R** | **I** | **S** | **I** | **S** | **S** | **S** | **R** | **S** |
| **G11** | **September 2018** | **Warriewood** | **ESBL-EC** | **R** | **R** | **R** | **R** | **R** | **R** | **S** | **R** | **R** | **R** | **S** | **S** | **S** | **R** | **S** | **S** | **S** | **R** | **S** |
| **G15** | **February 2019** | **Winmalee** | **ESBL-EC** | **R** | **R** | **R** | **R** | **R** | **R** | **S** | **S** | **S** | **S** | **R** | **I** | **S** | **S** | **S** | **R** | **R** | **R** | **I** |
| **G17** | **June 2019** | **Warriewood** | **ESBL-EC** | **R** | **R** | **R** | **R** | **R** | **R** | **S** | **R** | **I** | **R** | **R** | **S** | **S** | **R** | **S** | **R** | **R** | **R** | **S** |
|  | July 2017 | Wallacia | ESBL-EC | R | S | R | R | R | R | S | I | S | S | R | R | S | R | R | R | R | S | S |
|  | July 2017 | West Camden | ESBL-EC | R | S | R | S | R | S | S | S | S | S | R | R | S | R | R | R | R | S | S |
|  | July 2017 | Wollongong | ESBL-EC | R | R | R | R | R | S | S | R | S | S | S | I | S | S | S | R | R | S | S |
|  | July 2017 | Winmalee | ESBL-EC | R | I | R | R | R | R | S | S | S | S | S | S | S | R | R | S | S | S | S |
|  | July 2017 | Liverpool | ESBL-EC | R | S | R | R | R | S | S | I | S | S | S | R | S | S | S | R | R | S | S |
|  | July 2017 | Richmond | ESBL-EC | R | S | R | S | R | S | S | S | S | S | R | I | S | S | S | R | R | S | S |
|  | July 2017 | Hornsby Heights | ESBL-EC | R | S | R | S | R | S | S | S | S | S | R | S | S | S | S | R | R | S | S |
|  | July 2017 | Warriewood | ESBL-EC | R | S | R | R | R | R | S | S | S | S | S | S | S | S | S | S | S | S | S |
|  | July 2017 | Bombo | ESBL-EC | R | I | R | R | R | S | S | R | S | S | S | S | S | S | S | S | S | S | S |
|  | July 2017 | Brooklyn | ESBL-EC | R | S | R | S | R | S | S | R | I | S | S | S | S | S | S | S | S | S | S |
|  | July 2017 | Bondi | ESBL-EC | R | S | R | R | R | S | S | S | S | S | S | S | S | S | S | S | S | S | S |
|  | July 2017 | Picton | ESBL-EC | R | S | R | R | R | R | S | R | R | S | S | S | S | S | S | R | R | S | S |
|  | July 2017 | North Richmond | ESBL-EC | R | S | R | R | R | S | S | S | S | S | S | S | S | S | S | S | S | S | S |
|  | July 2017 | Quakers Hill | ESBL-EC | R | I | R | R | R | S | S | S | S | S | S | S | S | S | S | S | S | S | S |
|  | July 2017 | Malabar | ESBL-EC | R | S | R | S | R | S | S | S | S | S | S | S | S | S | S | R | R | S | S |
|  | July 2017 | St Marys | ESBL-EC | R | I | R | S | R | S | S | S | S | S | S | S | S | S | S | R | R | S | S |
|  | July 2017 | North Head | ESBL-EC | R | S | R | S | R | S | S | S | S | S | S | S | S | S | S | R | R | S | S |
|  | July 2017 | Penrith | ESBL-EC | R | S | R | S | R | S | S | S | S | S | S | S | S | S | S | S | S | S | S |
|  | July 2017 | Glenfield | ESBL-EC | R | S | R | S | R | S | S | S | S | S | S | S | S | S | S | S | S | S | S |
|  | July 2017 | Rouse Hill | ESBL-EC | R | S | R | S | R | S | S | S | S | S | S | S | S | S | S | S | S | S | S |
|  | July 2017 | Riverstone | ESBL-EC | R | S | R | S | R | S | S | S | S | S | S | S | S | S | S | S | S | S | S |
|  | July 2017 | Castle Hill | ESBL-EC | R | S | R | S | R | S | S | S | S | S | S | S | S | S | S | S | S | S | S |
|  | July 2017 | West Hornsby | ESBL-EC | R | S | R | S | R | S | S | S | S | S | S | S | S | S | S | S | S | S | S |
|  | July 2017 | Cronulla | ESBL-EC | R | S | R | S | R | S | S | S | S | S | S | S | S | S | S | S | S | S | S |
|  | February 2018 | Wollongong | ESBL-EC | R | R | R | R | R | R | R | R | R | R | S | S | S | R | R | R | R | S | S |
|  | February 2018 | Shellharbour | ESBL-EC | R | S | R | R | R | S | S | I | S | I | S | S | S | I | S | S | S | I | S |
|  | February 2018 | Penrith | ESBL-EC | R | S | R | R | R | R | S | S | S | S | R | I | S | S | S | R | R | I | S |
|  | February 2018 | Warriewood | ESBL-EC | R | S | R | R | R | R | S | S | S | S | R | I | S | R | R | R | R | S | S |
|  | February 2018 | Bondi | ESBL-EC | R | S | R | S | R | S | S | S | S | S | S | S | S | R | R | S | S | S | S |
|  | February 2018 | Bombo | ESBL-EC | R | S | R | R | R | R | S | R | S | R | S | S | S | R | R | S | S | S | S |
|  | February 2018 | West Hornsby | ESBL-EC | R | R | R | R | R | S | S | R | S | S | R | I | S | S | S | R | R | S | S |
|  | February 2018 | West Camden | ESBL-EC | R | S | R | S | R | S | S | R | S | S | S | S | S | I | S | R | R | S | S |
|  | February 2018 | St Marys | ESBL-EC | R | S | R | S | R | S | S | S | S | S | S | S | S | S | S | R | R | S | S |
|  | February 2018 | North Richmond | ESBL-EC | R | S | R | S | R | S | S | S | S | S | S | S | S | S | S | R | R | S | S |
|  | February 2018 | Richmond | ESBL-EC | R | S | R | S | R | S | S | S | S | S | S | S | S | S | S | R | R | S | S |
|  | February 2018 | Riverstone | ESBL-EC | R | S | R | R | R | R | S | S | S | S | S | S | S | I | S | R | R | S | S |
|  | February 2018 | Quakers Hill | ESBL-EC | R | S | R | R | R | R | S | R | I | R | R | I | S | S | S | R | R | S | S |
|  | February 2018 | North Head | ESBL-EC | R | I | R | R | R | R | S | I | S | R | R | I | S | S | S | R | R | S | S |
|  | February 2018 | Picton | ESBL-EC | R | S | R | R | R | R | S | S | S | S | S | S | S | I | S | R | R | S | S |
|  | February 2018 | Liverpool | ESBL-EC | R | S | R | S | R | S | S | S | S | S | R | I | S | S | S | S | S | S | S |
|  | February 2018 | Glenfield | ESBL-EC | R | S | R | R | R | S | S | S | S | S | S | S | S | S | S | S | S | S | S |
|  | February 2018 | Malabar | ESBL-EC | R | S | R | S | R | S | S | S | S | S | S | S | S | S | S | S | S | S | S |
|  | February 2018 | North Head | ESBL-EC | R | S | R | S | R | S | S | S | S | S | S | S | S | S | S | S | S | S | S |
|  | February 2018 | Castle Hill | ESBL-EC | R | S | R | S | R | S | S | S | S | S | S | S | S | S | S | S | S | S | S |
|  | February 2018 | Rouse Hill | ESBL-EC | R | S | R | S | R | S | S | S | S | S | S | S | S | S | S | S | S | S | S |
|  | May 2018 | Liverpool | ESBL-EC | R | R | R | R | R | I | R | R | R | S | S | S | S | R | R | R | R | S | S |
|  | May 2018 | Bondi | ESBL-EC | R | R | R | R | R | I | S | R | I | S | R | S | S | R | R | R | R | I | S |
|  | May 2018 | Bombo | ESBL-EC | R | R | R | S | R | S | S | I | S | S | R | I | S | R | R | R | R | S | S |
|  | May 2018 | Riverstone | ESBL-EC | R | R | R | R | R | S | S | R | S | S | S | S | S | S | S | R | R | S | S |
|  | May 2018 | Picton | ESBL-EC | R | R | R | R | R | S | S | R | S | S | R | I | S | S | S | R | R | S | S |
|  | May 2018 | Malabar | ESBL-EC | R | R | R | R | R | R | S | R | I | S | S | S | S | S | S | S | S | S | S |
|  | May 2018 | Rouse Hill | ESBL-EC | R | S | R | R | R | I | S | I | S | S | R | R | S | R | R | S | S | S | S |
|  | May 2018 | North Head | ESBL-EC | R | S | R | R | R | S | S | S | S | S | S | S | S | R | R | S | S | S | S |
|  | May 2018 | West Camden | ESBL-EC | R | S | R | R | R | S | S | S | S | S | S | S | S | R | R | S | S | S | S |
|  | May 2018 | Rouse Hill | ESBL-EC | R | S | R | R | R | S | S | I | S | S | S | S | S | R | R | S | S | S | S |
|  | May 2018 | Hornsby Heights | ESBL-EC | R | S | R | R | R | S | S | S | S | S | S | S | S | R | R | R | R | I | S |
|  | May 2018 | West Hornsby | ESBL-EC | R | S | R | S | R | S | S | I | S | S | S | S | S | R | R | R | R | S | S |
|  | May 2018 | Shellharbour | ESBL-EC | R | S | R | S | R | S | S | S | S | S | S | S | S | R | R | R | R | S | S |
|  | May 2018 | North Richmond | ESBL-EC | R | S | R | R | R | S | S | S | S | S | S | S | S | S | S | R | R | S | S |
|  | May 2018 | Wollongong | ESBL-EC | R | I | R | S | R | S | S | S | S | S | S | S | S | S | S | R | R | I | S |
|  | May 2018 | St Marys | ESBL-EC | R | S | R | S | R | S | S | S | S | S | S | S | S | S | S | R | S | S | S |
|  | May 2018 | Brooklyn | ESBL-EC | R | S | R | S | R | S | S | S | S | S | R | I | S | S | S | S | S | S | S |
|  | May 2018 | Cronulla | ESBL-EC | R | S | R | R | R | I | S | S | S | S | S | S | S | S | S | S | S | S | S |
|  | May 2018 | Glenfield | ESBL-EC | R | S | R | R | R | S | S | S | S | S | S | S | S | S | S | S | S | S | S |
|  | May 2018 | Penrith | ESBL-EC | R | I | R | S | R | S | S | S | S | S | S | S | S | S | S | S | S | S | S |
|  | May 2018 | Wallacia | ESBL-EC | R | S | R | S | R | S | S | S | S | S | S | S | S | S | S | S | S | S | S |
|  | May 2018 | Richmond | ESBL-EC | R | S | R | S | R | S | S | S | S | S | S | S | S | S | S | S | S | S | S |
|  | May 2018 | Winmalee | ESBL-EC | R | S | R | S | R | S | S | S | S | S | S | S | S | S | S | S | S | S | S |
|  | May 2018 | Warriewood | ESBL-EC | R | S | R | S | R | S | S | S | S | S | S | S | S | S | S | S | S | S | S |
|  | September 2018 | Penrith | ESBL-EC | R | S | R | R | R | S | R | S | R | I | R | S | R | S | S | S | S | S | S |
|  | September 2018 | West Hornsby | ESBL-EC | R | R | R | R | R | R | S | R | S | R | S | R | S | R | R | R | R | I | S |
|  | September 2018 | Riverstone | ESBL-EC | R | S | R | R | R | S | S | I | S | I | S | R | S | R | R | R | R | S | S |
|  | September 2018 | Quakers Hill | ESBL-EC | R | S | R | R | R | R | S | S | S | S | R | I | S | R | R | R | R | S | S |
|  | September 2018 | St Marys | ESBL-EC | R | R | R | R | R | R | S | S | S | I | S | S | S | R | R | S | S | S | S |
|  | September 2018 | Hornsby Heights | ESBL-EC | R | R | R | R | R | R | S | S | S | S | S | S | S | R | R | S | S | I | S |
|  | September 2018 | Glenfield | ESBL-EC | R | R | R | R | R | R | S | R | S | S | S | S | S | S | S | R | S | S | S |
|  | September 2018 | Cronulla | ESBL-EC | R | S | R | R | R | R | S | S | S | S | S | S | S | R | R | R | R | S | S |
|  | September 2018 | Liverpool | ESBL-EC | R | I | R | R | R | R | S | S | S | I | S | S | S | R | R | R | R | I | S |
|  | September 2018 | Wollongong | ESBL-EC | R | S | R | R | R | R | S | S | S | S | R | I | S | R | R | S | S | S | S |
|  | September 2018 | Wallacia | ESBL-EC | R | S | R | R | R | R | S | S | S | S | R | S | S | S | S | R | S | S | S |
|  | September 2018 | Brooklyn | ESBL-EC | R | S | R | R | R | R | S | I | S | I | R | I | S | S | S | R | R | S | S |
|  | September 2018 | Picton | ESBL-EC | R | S | R | R | R | R | S | I | S | I | R | S | S | S | S | R | R | S | S |
|  | September 2018 | Penrith | ESBL-EC | R | S | R | R | R | R | S | S | S | S | S | S | S | S | S | R | R | S | S |
|  | September 2018 | North Richmond | ESBL-EC | R | S | R | R | R | R | S | S | S | S | S | S | S | S | S | R | R | S | S |
|  | September 2018 | Richmond | ESBL-EC | R | S | R | R | R | R | S | S | S | S | S | S | S | I | S | R | R | S | S |
|  | September 2018 | Malabar | ESBL-EC | R | S | R | R | R | R | S | S | S | S | S | S | S | S | S | S | S | S | S |
|  | September 2018 | Bondi | ESBL-EC | R | S | R | R | R | R | S | S | S | S | S | S | S | S | S | S | S | S | S |
|  | September 2018 | Winmalee | ESBL-EC | R | S | R | R | R | R | S | I | S | I | S | S | S | S | S | S | S | S | S |
|  | September 2018 | North Head | ESBL-EC | R | S | R | R | R | R | S | I | S | I | S | S | S | S | S | S | S | S | S |
|  | September 2018 | Shellharbour | ESBL-EC | R | S | R | R | R | R | S | S | S | S | S | S | S | S | S | S | S | S | S |
|  | September 2018 | Bombo | ESBL-EC | R | S | R | R | R | R | S | S | S | S | S | S | S | S | S | S | S | S | S |
|  | September 2018 | West Camden | ESBL-EC | R | S | R | R | R | R | S | S | S | I | S | I | S | S | S | S | S | S | S |
|  | September 2018 | Rouse Hill | ESBL-EC | R | S | R | R | R | R | S | S | S | S | S | S | S | S | S | S | S | S | S |
|  | February 2019 | Riverstone | ESBL-EC | R | R | R | R | R | R | S | R | S | R | R | R | S | R | R | R | R | R | S |
|  | February 2019 | North Richmond | ESBL-EC | R | S | R | R | R | R | S | S | S | S | R | I | S | R | R | R | R | S | S |
|  | February 2019 | Winmalee | ESBL-EC | R | I | R | R | R | R | S | I | S | I | R | I | S | R | S | R | R | S | S |
|  | February 2019 | Cronulla | ESBL-EC | R | S | R | R | R | R | S | S | S | S | R | I | S | R | S | R | R | S | S |
|  | February 2019 | Richmond | ESBL-EC | R | S | R | R | R | R | S | R | S | S | S | S | S | R | R | R | R | S | S |
|  | February 2019 | Richmond | ESBL-EC | R | S | R | R | R | R | S | R | S | S | S | S | S | R | R | R | R | S | S |
|  | February 2019 | Castle Hill | ESBL-EC | R | R | R | R | R | R | S | R | I | R | S | S | S | R | R | R | R | S | S |
|  | February 2019 | Picton | ESBL-EC | R | S | R | R | R | R | S | S | I | S | S | S | S | R | R | R | R | S | S |
|  | February 2019 | Bondi | ESBL-EC | R | S | R | R | R | R | S | S | S | S | S | S | S | S | S | R | S | S | S |
|  | February 2019 | North Head | ESBL-EC | R | S | R | R | R | R | S | S | S | S | S | S | S | R | S | R | R | S | S |
|  | February 2019 | Brooklyn | ESBL-EC | R | R | R | R | R | R | S | R | S | S | S | S | S | R | S | R | R | S | S |
|  | February 2019 | West Hornsby | ESBL-EC | R | S | R | R | R | R | S | R | S | R | S | S | S | R | S | R | R | S | S |
|  | February 2019 | Warriewood | ESBL-EC | R | S | R | R | R | R | S | S | S | I | S | S | S | R | R | S | S | S | S |
|  | February 2019 | Rouse Hill | ESBL-EC | R | S | R | R | R | R | S | S | S | S | S | S | S | R | R | S | S | S | S |
|  | February 2019 | Malabar | ESBL-EC | R | S | R | R | R | R | S | S | S | S | S | S | S | I | S | R | R | S | S |
|  | February 2019 | Malabar | ESBL-EC | R | S | R | R | R | R | S | I | S | I | S | S | S | S | S | R | R | S | S |
|  | February 2019 | Wollongong | ESBL-EC | R | S | R | R | R | R | S | S | S | S | S | S | S | S | S | R | R | S | S |
|  | February 2019 | Penrith | ESBL-EC | R | S | R | R | R | R | S | I | S | I | S | S | S | S | S | R | R | I | S |
|  | February 2019 | Hornsby Heights | ESBL-EC | R | S | R | R | R | R | S | I | S | I | S | S | S | S | S | R | R | S | S |
|  | February 2019 | Hornsby Heights | ESBL-EC | R | S | R | R | R | R | S | S | S | S | S | S | S | S | S | R | R | S | S |
|  | February 2019 | Liverpool | ESBL-EC | R | S | R | R | R | R | S | I | S | I | S | S | S | S | S | R | R | S | S |
|  | February 2019 | Wallacia | ESBL-EC | R | S | R | R | R | R | S | S | S | S | R | I | S | I | S | S | S | S | S |
|  | February 2019 | St Marys | ESBL-EC | R | S | R | R | R | R | S | R | R | R | S | S | S | S | S | S | S | S | S |
|  | February 2019 | Warriewood | ESBL-EC | R | S | R | R | R | R | S | S | S | S | S | S | S | S | S | S | S | S | S |
|  | February 2019 | Shellharbour | ESBL-EC | R | S | R | R | R | R | S | I | S | I | S | S | S | S | S | S | S | S | S |
|  | February 2019 | Bombo | ESBL-EC | R | S | R | R | R | R | S | S | S | S | S | S | S | S | S | S | S | S | S |
|  | February 2019 | Penrith | ESBL-EC | R | S | R | R | R | R | S | S | S | S | S | S | S | S | S | S | S | S | S |
|  | February 2019 | Quakers Hill | ESBL-EC | R | S | R | R | R | R | S | S | S | S | S | S | S | S | S | S | S | S | S |
|  | February 2019 | St Marys | ESBL-EC | R | S | R | R | R | R | S | S | S | I | S | S | S | S | S | S | S | S | S |
|  | February 2019 | West Camden | ESBL-EC | R | S | R | R | R | R | S | S | S | I | S | S | S | S | S | S | S | S | S |
|  | June 2019 | Shellharbour | ESBL-EC | R | S | R | R | R | R | S | I | S | I | R | I | S | R | R | S | S | S | S |
|  | June 2019 | West Camden | ESBL-EC | R | R | R | R | R | R | S | R | R | R | S | S | S | R | R | S | S | S | S |
|  | June 2019 | North Head | ESBL-EC | R | S | R | R | R | R | S | I | S | I | S | S | S | R | R | S | S | S | S |
|  | June 2019 | Castle Hill | ESBL-EC | R | S | R | R | R | R | S | S | S | S | S | S | S | R | R | S | S | S | S |
|  | June 2019 | Hornsby Heights | ESBL-EC | R | S | R | R | R | R | S | S | S | S | S | S | S | R | S | R | R | S | S |
|  | June 2019 | Winmalee | ESBL-EC | R | S | R | R | R | R | S | I | S | I | R | I | S | I | S | R | R | S | S |
|  | June 2019 | Picton | ESBL-EC | R | S | R | R | R | R | S | S | S | S | R | I | S | S | S | R | R | S | S |
|  | June 2019 | Malabar | ESBL-EC | R | S | R | R | R | R | S | R | R | R | R | R | S | S | S | R | R | S | S |
|  | June 2019 | Wallacia | ESBL-EC | R | R | R | R | R | R | S | R | R | R | S | S | S | S | S | R | R | S | S |
|  | June 2019 | Glenfield | ESBL-EC | R | S | R | R | R | R | S | S | S | S | S | S | S | S | S | R | R | S | S |
|  | June 2019 | St Marys | ESBL-EC | R | S | R | R | R | R | S | S | S | S | S | S | S | S | S | R | R | S | S |
|  | June 2019 | Bondi | ESBL-EC | R | S | R | R | R | R | S | S | S | I | S | S | S | R | S | S | S | S | S |
|  | June 2019 | Richmond | ESBL-EC | R | I | R | R | R | R | S | R | S | I | S | S | S | S | S | S | S | S | S |
|  | June 2019 | Liverpool | ESBL-EC | R | S | R | R | R | R | S | S | S | S | S | S | S | S | S | S | S | S | S |
|  | June 2019 | North Richmond | ESBL-EC | R | I | R | R | R | R | S | R | S | I | S | S | S | S | S | S | S | S | S |
|  | June 2019 | Brooklyn | ESBL-EC | R | R | R | R | R | R | S | R | R | R | S | S | S | S | S | S | S | S | S |
|  | June 2019 | West Hornsby | ESBL-EC | R | S | R | R | R | R | S | S | S | S | S | S | S | S | S | S | S | S | S |
|  | June 2019 | Cronulla | ESBL-EC | R | S | R | R | R | R | S | S | S | S | S | S | S | S | S | S | S | S | S |
|  | June 2019 | Quakers Hill | ESBL-EC | R | R | R | R | R | R | S | R | I | R | S | S | S | S | S | S | S | S | S |
|  | June 2019 | Penrith | ESBL-EC | R | R | R | R | R | R | S | R | S | R | S | S | S | S | S | S | S | S | S |
|  | June 2019 | Bombo | ESBL-EC | R | S | R | R | R | R | S | S | S | S | S | S | S | S | S | S | S | S | S |
|  | June 2019 | Rouse Hill | ESBL-EC | R | S | R | R | R | R | S | S | S | S | S | S | S | S | S | S | S | S | S |
|  | June 2019 | Riverstone | ESBL-EC | R | S | R | R | R | R | S | S | S | S | S | S | S | S | S | S | S | S | S |
|  | June 2019 | Wollongong | ESBL-EC | R | S | R | R | R | R | S | S | I | S | S | S | S | S | S | S | S | S | S |
| **Categories of important antibiotics for human medicine (WHO, 2019)** | | | | Critically important (High priority) | Highly important | Highly important | Critically important (Highest priority) | Critically important (Highest priority) | Critically important (Highest priority) | Critically important (High priority) | Critically important (High priority) | Critically important (High priority) | Not available | Critically important (High priority) | Critically important (High priority) | Critically important (High priority) | Critically important (Highest priority) | Critically important (Highest priority) | Highly important | Highly important | Important | Critically important (High priority) |

**Table S2.** General characteristics of assembled *E. coli* genomes.

| Genomes | Number of contigs | N50 (Kbp) | Approximate genome size (Mbp) | G + C content (%) | Number of predicted genes | Number of predicted protein-coding sequences (CDS) |
| --- | --- | --- | --- | --- | --- | --- |
| G4 | 469 | 76.68 | 6.03 | 51.45 | 5,951 | 5,882 |
| G5 | 270 | 60.83 | 6.13 | 51.81 | 6,002 | 5,943 |
| G8 | 115 | 130.61 | 5.00 | 50.58 | 4,742 | 4,676 |
| G16 | 95 | 191.17 | 4.82 | 50.53 | 4,562 | 4,498 |
| G10 | 93 | 134.57 | 4.87 | 50.81 | 4,638 | 4,573 |
| G11 | 86 | 198.10 | 5.07 | 50.46 | 4,790 | 4,721 |
| G15 | 73 | 213.40 | 5.02 | 50.56 | 4,729 | 4,664 |
| G17 | 116 | 191.08 | 5.10 | 50.52 | 4,826 | 4,759 |

**Table S3.** Analysis of mutations identified in the OmpC porin of CR-EC and ESBL-EC isolates.

| **Isolates** | **Protein** | **Functional impact of mutations predicted by PROVEAN** | |
| --- | --- | --- | --- |
|  |  | **Neutral** | **Deleterious** |
| G4, G5, G8 | OmpC | V24I, K48D, D49S, V50K, Q54K, S85E, A86P, N88S, E89D, N91S, G137D, N176S, P177A, S178H, F182M, S184T, N189G, A193V, L194F, R195E, G216A, I218V, A226D, T229N, A230T, A231G, Y232L, N235T, Q346R, N357D, deletions of GVT at positions 185-187 (del G-T (185-T187)) | N165D, G190R, R191D, D208N, D225W |
| G16 | OmpC | N176S, P177V, V186M, T229S, A230P, A231L, L296V, G309N, del FTSG (182-185) and insertions of GTIA between positions 306-307 (Ins GTIA (182-185)) | D192G, R195L |
| G10 | OmpC | D49S, V50E, M57V, S85T, A86S, E89K, N90E, F149Y, N176S, P177V, V186M, G216A, I218V, A226D, T229F, A230E, A231R, I233L, L296V, G309N, Ins D (87-88), del FTSG (182-185), Ins VVAG (307-308) | D192G, Ins GLNGYG (229-230) |
| G11, G15, G17 | OmpC | D49S, V50E, N176S, P177V, S178D, V186M, G216A, I218V, T229F, A230E, A231R, I233L, L296V, G309N, Q346R, N357D, del FTSG (182-185), Ins VING (307-308) | D192G, Ins GLNGYG (229-230) |

**Table S4.** List of contigs co-harbouring antibiotic resistance and virulence genes in the assembled genome of *E. coli* isolates from wastewater.

| **Isolates** | **Contig (size bp)** | **Plasmid replicon (position in contig)** | **Resistance gene (position in contig)** | **Virulence gene (position in contig)** |
| --- | --- | --- | --- | --- |
| G4 | Node_74 (9606) | IncQ2 (4852 to 5301) | *qnrS2* (2564 to 3220) | Not detected (ND) |
| G5 | Node_27 (61430) | IncM2 (1921 to 2584) | *bla*_TEM-1B_ (60585 to 61355) | ND |
| G16 | Node_51 (4540) | IncQ1 (3940 to 4468) | *sul2* (1786 to 2601) | ND |
| G10 | Node_56 (10794) | IncFIB(AP001918) (1719 to 2400) | ND | *hlyF* (5454 to 6563), *ompT* (6996 to 7949) |
| G11 | Node_42 (10524) | Col156 (6753 to 6894) | ND | *senB* (948 to 2123) |
| G15 | Node_31 (19297) | IncFII(29) (6175 to 6433) | *bla*_TEM-1B_ (313 to 1173) | ND |
|  | Node_45 (10524) | Col156 (6753 to 6894) | ND | *senB* (948 to 2123) |
| G17 | Node_43 (10524) | Col156 (3631 to 3772) | ND | *senB* (8402 to 9577) |

**Table S5.** Impact of mutations in the genes encoding proteins related to survival and motility of the wastewater *E. coli* isolates.

| **Isolates** | **Protein** | **Functional impact of mutations predicted by PROVEAN** | |
| --- | --- | --- | --- |
|  |  | **Neutral** | **Deleterious** |
| G4 | NfrA | A557T, T603A | Del A-L (598-602) |
| G11 | NfrA | E393G, S511N, A533T, K573Q, S578N, V628I, P671Q, I784L, I835L | Del M-T (1-308) |
| G16 | NfrA | R101Q, I274N, A357P, A364S, S511N, K573Q, S578N, N600S, T648I, P671Q, I835L | N645T |
| G4, G5, G8 | Cir | D95Q, S96Q | S90Y, I91S, R92W, G93S, L94G, S97L, Y98H, T99P, L100D, I101S, L102R, V103R, D104R, del K-F (106-663) |
| G10 | Cir | G161R, Q162S, K163E, S165V, T169Y, V170R, T172Y, R178S, D179R, R180S | I160N, W164M, T167Y, V168R, D171R, T173H, I174H, Q175S, E176G, H177T, G181R, del D-F (182-663) |
| G4, G5, G8 | FlhA | L30M | Del M-L (1-29), I31S, L32N, S33L, M34A, M35A, V36M, P38R |
| G4, G5, G8 | FliI | Q45R | L46N, P47L, L48C, G49H, del A-S (50-457) |
| G10 | FliP | I217L | A215P, T216P, A218L, L219C, F221L, K222N, del L-S (223-245) |


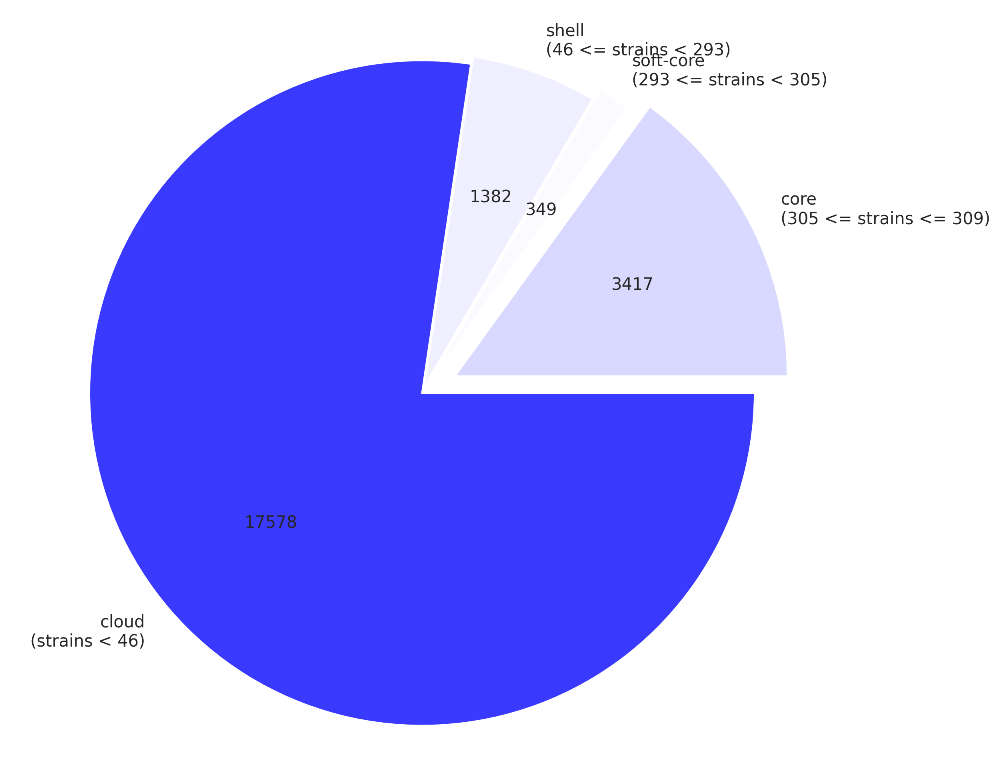


**Fig. S1.** Distribution of pan genes, core genes, and soft core genes among the 309 *E. coli* isolates belonging to ST10 clonal complex identified by Roary.


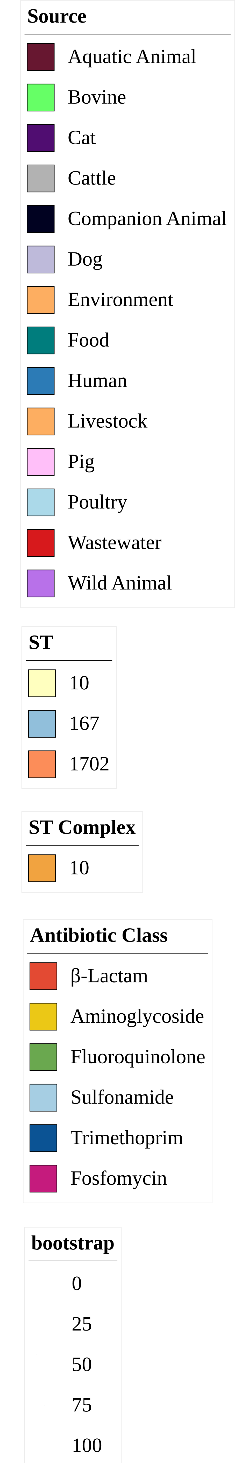




**(Fig. S3)**

**Fig. S2.** Maximum likelihood phylogeny of 309 ST10, ST167, and ST1702 *E. coli* isolates of the ST10 clonal complex inferred from the alignment of the concatenated core CDS (n=3,417) determined by Roary and RAxML using the GTR-Gamma model and 100 bootstrap iterations. The tree is midpoint rooted, and the tree nodes are labeled with the origin, year, and accession number of the isolates. The wastewater isolates are in bold and highlighted in pink for ST167 and blue for ST1702. The remaining Australian isolates from Enterobase are in bold. The source of the isolates, sequence types (ST), ST complexes, and acquired resistance genes against different clinically important antibiotic classes are annotated on the colored strips according to the given key. For ease of visualization and analysis, the tree was pruned at the clade (Fig. S3) containing wastewater isolates.


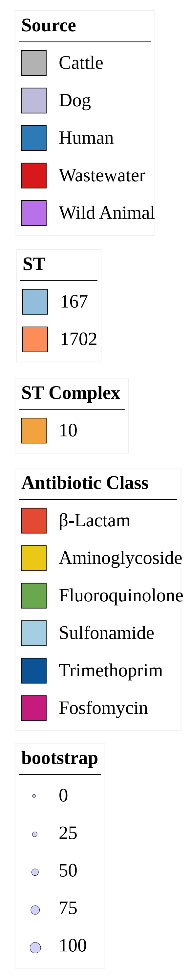




**Fig. S3.** Maximum-likelihood phylogeny pruned from a large phylogeny of 309 ST10, ST167, and ST1702 *E. coli* isolates of the ST10 clonal complex (Fig. S2). The phylogeny was inferred from the alignment of the concatenated core CDS (n = 3,417) determined by Roary and RAxML using the GTR-Gamma model and 100 bootstrap iterations. The tree nodes are labeled with the origin, year of isolation, and accession number of the isolates. The wastewater isolates are in bold and highlighted in pink for ST167 and blue for ST1702. The remaining Australian isolates from Enterobase are in bold. The source of the isolates, sequence types (ST), ST complexes, and acquired resistance genes against clinically important antibiotic classes are annotated on the colored strips according to the given key.


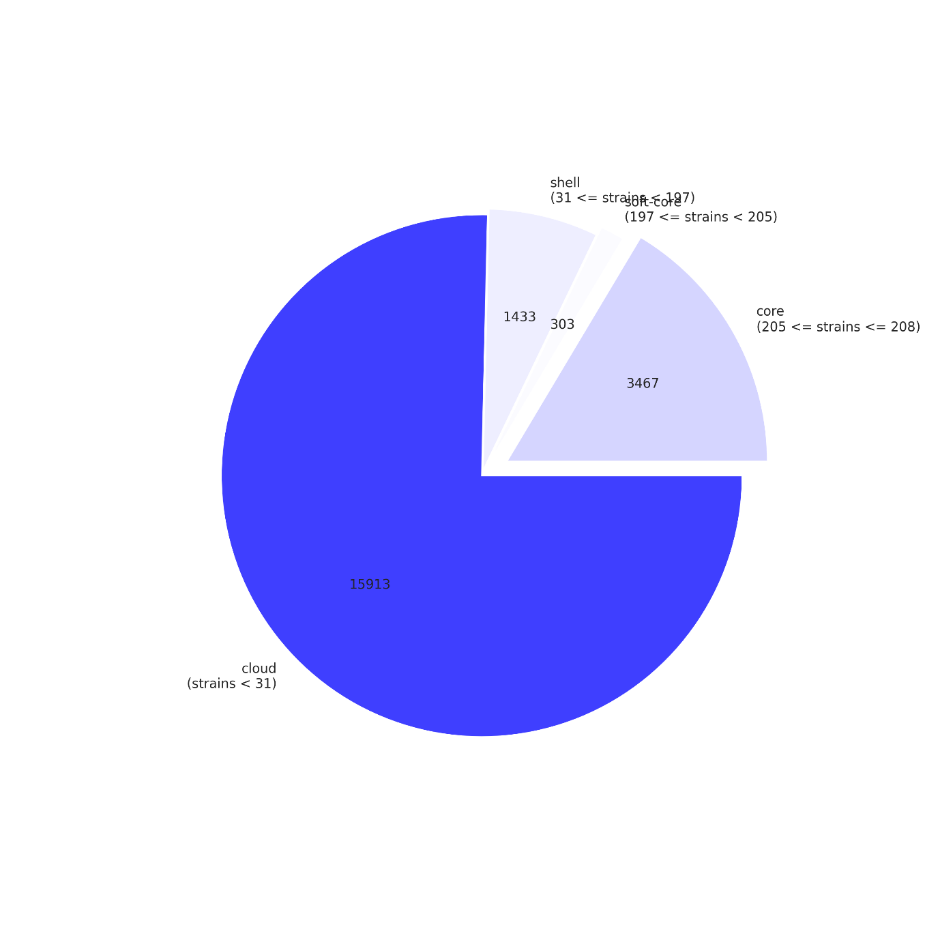


**Fig. S4**The distribution of pan genes, core genes, and soft-core genes among the 208 *E. coli* isolates belonging to the ST23 clonal complex identified by Roary.


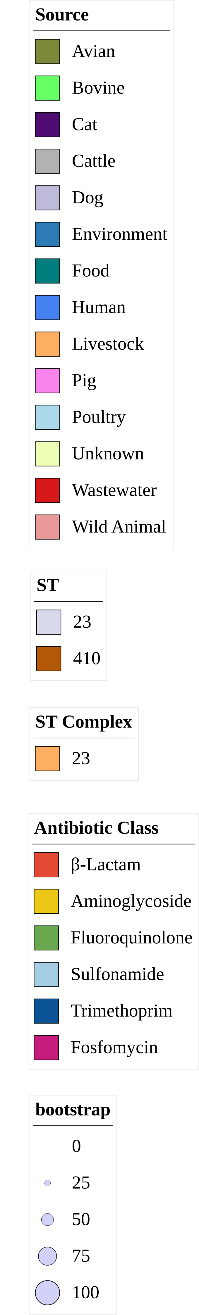




**Fig. S5.** Maximum likelihood phylogeny of 208 ST410 and closely related ST23 *E. coli* isolates of the ST23 clonal complex inferred from the alignment of the concatenated core CDS (n=3,467) determined by Roary and RAxML using the GTR-Gamma model and 100 bootstrap iterations. The tree is midpoint rooted, and the tree nodes are labeled with the origin, year, and accession number of the isolates. The wastewater isolates belonging to ST410 are in bold and highlighted in pink. ST23 isolates are highlighted in blue, and the remaining Australian isolates from Enterobase are in bold. The source of the isolates, sequence types (ST), ST complexes, and the acquired resistance genes against different clinically important antibiotic classes are annotated on the colored strips according to the given key. For ease of visualization and analysis, the tree was pruned at the clade containing wastewater isolates (see Fig. S6).


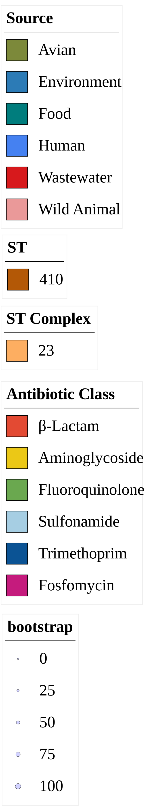

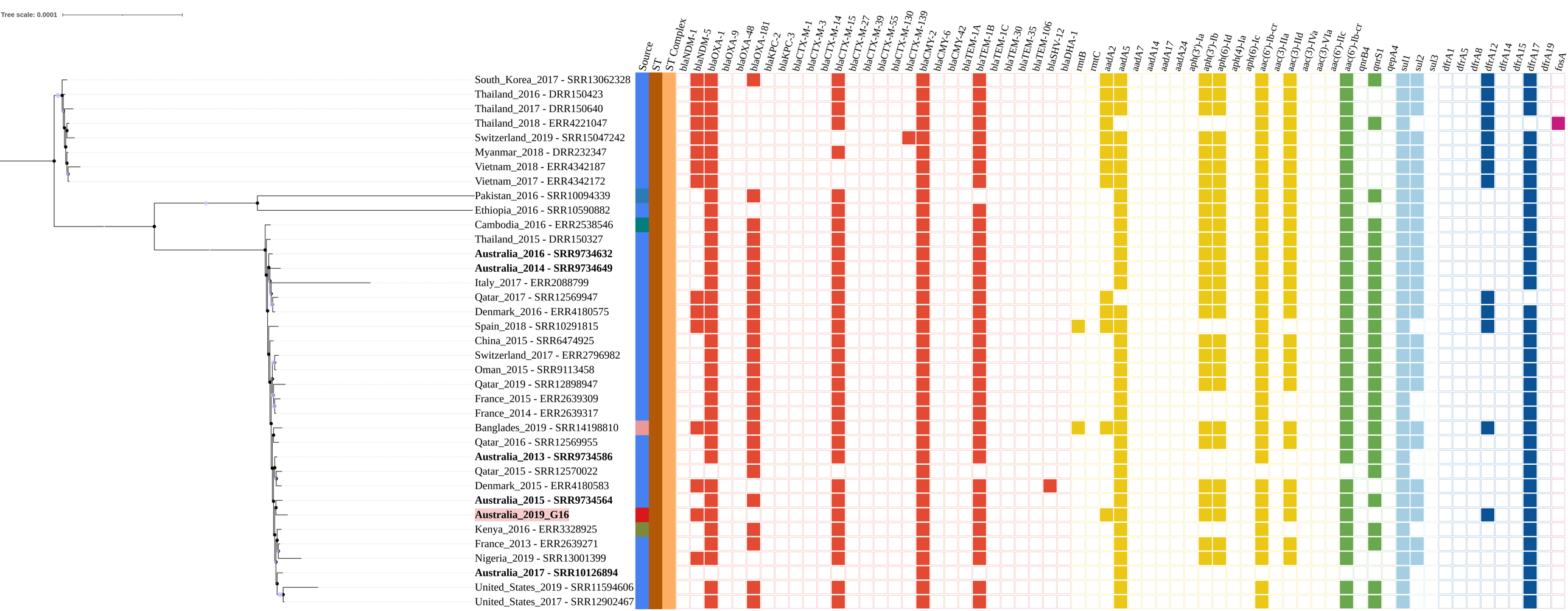


**Fig. S6**. Maximum-likelihood phylogeny pruned from a large phylogeny of 208 ST410 and ST23 *E. coli* of the ST23 clonal complex (Fig. S5)). The phylogeny was inferred from the alignment of the concatenated core CDS (n = 3,467) determined by Roary and RAxML using the GTR-Gamma model and 100 bootstrap iterations. The tree nodes are labeled with the origin, year of isolation, and accession number of the isolates. The wastewater isolate is in bold and highlighted in pink, and the remaining Australian isolates from Enterobase are in bold. The source of the isolates, sequence types (ST), ST complexes, and acquired resistance genes against different clinically important antibiotic classes are annotated on the colored strips according to the given key.


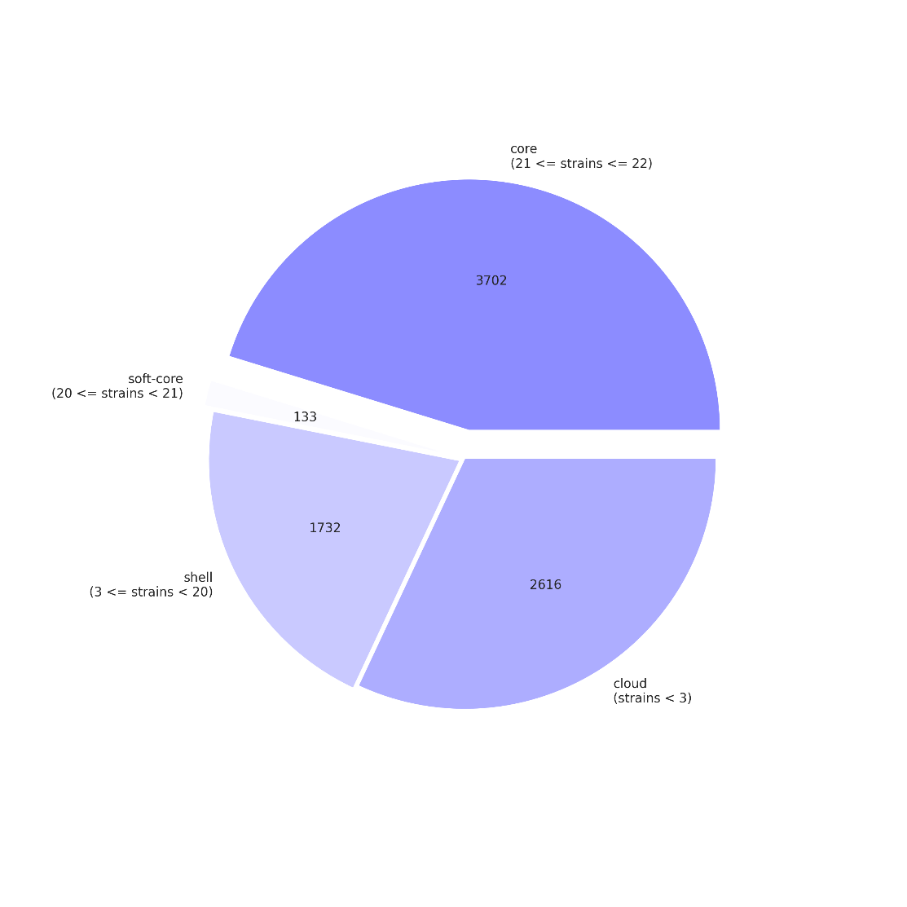


**Fig. S7.** The distribution of pan genes, core genes, and soft-core genes among the 22 *E. coli* isolates belonging to ST9586 and closely related ST678 clones identified by Roary.


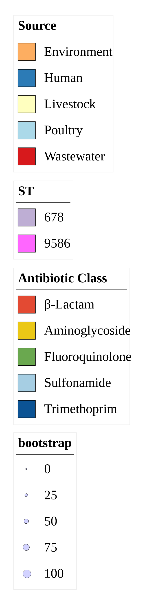




Fig. S8. The maximum likelihood phylogeny of ST9586 and closely related ST678 *E. coli* isolates (n = 22) was inferred from the alignment of the concatenated core CDS (n = 3,702) determined by Roary and RAxML using the GTR-Gamma model and 1,000 bootstrap iterations. The tree is midpoint rooted, and the tree nodes are labeled with the origin, year of isolation, and accession number of the isolates. The wastewater isolate is in bold and highlighted in pink. The source of the isolates, sequence types (ST), and acquired resistance genes against clinically important antibiotic classes are annotated on the colored strips according to the given key.


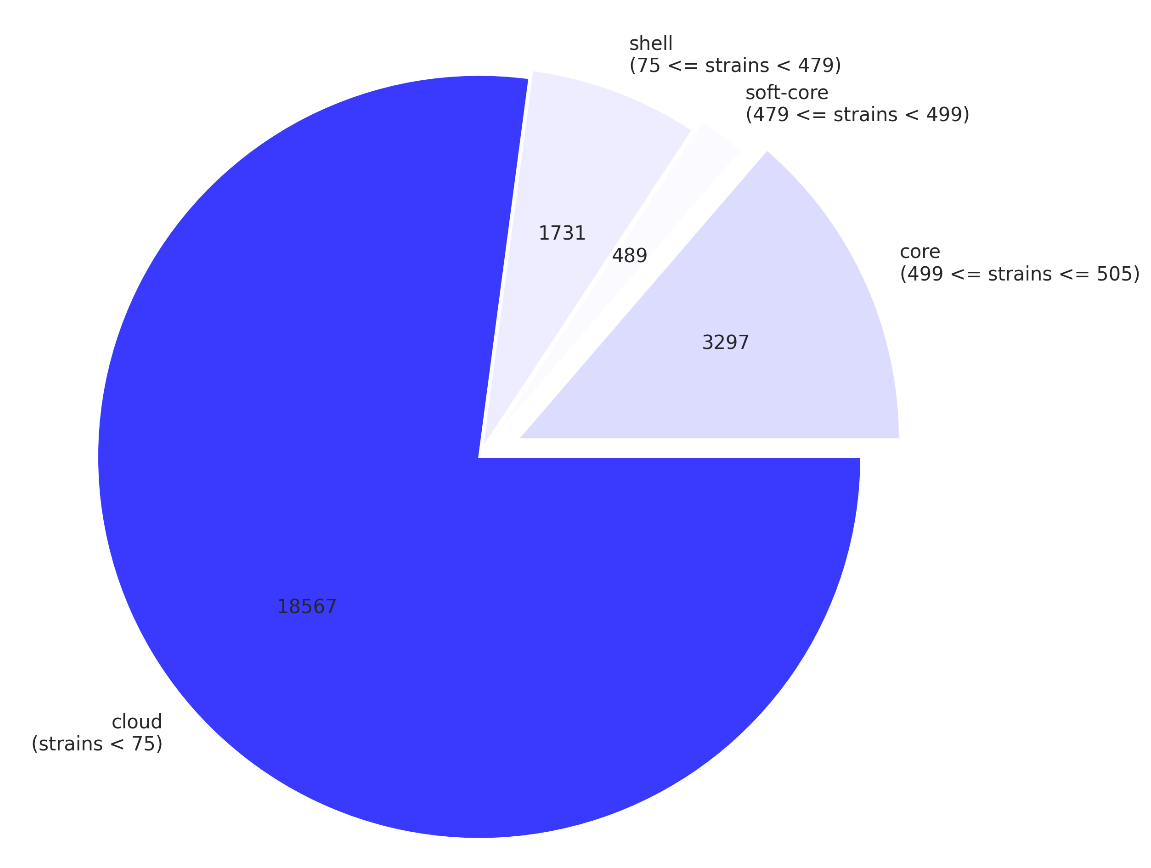


**Fig. S9.** The distribution of pan genes, core genes, and soft-core genes among the 505 *E. coli* isolates belonging to the ST131 clonal complex identified by Roary.


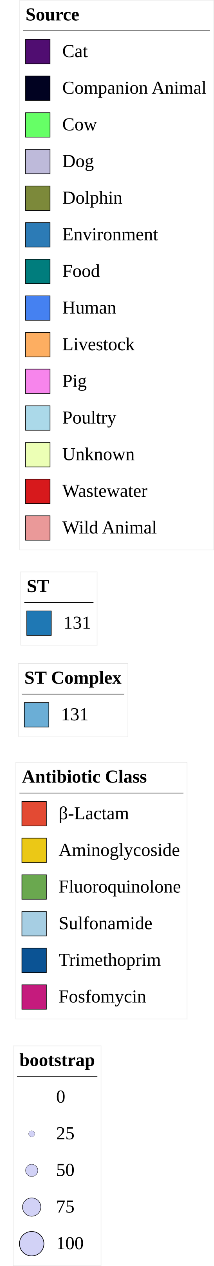




**(Fig.S11)**

**Fig. S10.** Maximum likelihood phylogeny of 505 ST131 *E. coli* isolates inferred from the alignment of the concatenated core CDS (n=3,297) determined by Roary and RAxML using the GTR-Gamma model and 100 bootstrap iterations. The tree is midpoint rooted, and the nodes are labeled with the origin, year, and accession number of the isolates. Three wastewater isolates belonging to ST131 are in bold and highlighted in pink. The remaining Australian isolates from Enterobase are in bold. The source of the isolates, sequence types (ST), ST complexes, and the acquired resistance genes against different clinically important antibiotic classes are annotated on the colored strips according to the given key. For ease of visualization and analysis, the tree was pruned (Fig. S11) at the clade containing wastewater isolates.


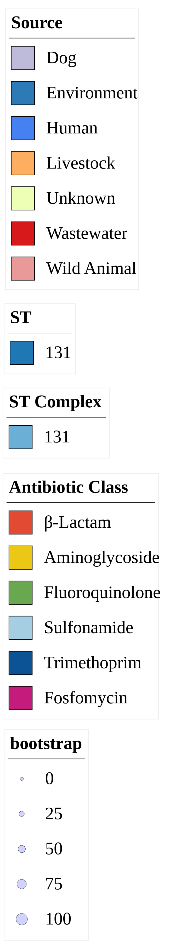




**Fig. S11.** Maximum-likelihood phylogeny pruned from a large phylogeny of 505 ST131 *E. coli* isolates of the ST131 clonal complex (Fig. S10). The phylogeny was inferred from the alignment of the concatenated core CDS (n = 3,297) determined by Roary and RAxML using the GTR-Gamma model and 100 bootstrap iterations. The tree nodes are labeled with the origin, year of isolation, and accession number of the isolates. The wastewater isolates are in bold and highlighted in pink, and the remaining Australian isolates from Enterobase are in bold. The source of the isolates, sequence types (ST), ST complexes, and acquired resistance genes against different clinically important antibiotic classes are annotated on the colored strips according to the given key.
